# Transplantation of induced neural stem cells (iNSCs) into chronically demyelinated corpus callosum ameliorates motor deficits

**DOI:** 10.1186/s40478-020-00960-3

**Published:** 2020-06-09

**Authors:** Genevieve M. Sullivan, Andrew K. Knutsen, Luca Peruzzotti-Jametti, Alexandru Korotcov, Asamoah Bosomtwi, Bernard J. Dardzinski, Joshua D. Bernstock, Sandra Rizzi, Frank Edenhofer, Stefano Pluchino, Regina C. Armstrong

**Affiliations:** 1 Department of Anatomy, Physiology and Genetics, F. Edward Hebert School of Medicine, Uniformed Services University of the Health Sciences, 4301 Jones Bridge Rd, Bethesda, MD 20814 USA; 2 Center for Neuroscience and Regenerative Medicine, F. Edward Hebert School of Medicine, Uniformed Services University of the Health Sciences, Bethesda, MD 20814 USA; 3 Department of Radiology and Radiological Sciences, F. Edward Hebert School of Medicine, Uniformed Services University of the Health Sciences, Bethesda, MD 20814 USA; 4 Department of Clinical Neurosciences and NIHR Biomedical Research Centre, University of Cambridge, Cambridge, UK; 5 Present address: Department of Neurosurgery, Brigham and Women’s Hospital, Harvard Medical School, Boston, MA USA; 6 Genomics, Stem Cell Biology and Regenerative Medicine & CMBI, Leopold-Franzens-University Innsbruck, Innsbruck, Austria

**Keywords:** Neural stem cells, Remyelination, Cuprizone, Multiple sclerosis, Stem cell therapeutics, Diffusion tensor imaging, Magnetization transfer ratio, Magnetic resonance imaging, Reprogramming

## Abstract

Multiple Sclerosis (MS) causes neurologic disability due to inflammation, demyelination, and neurodegeneration. Immunosuppressive treatments can modify the disease course but do not effectively promote remyelination or prevent long term neurodegeneration. As a novel approach to mitigate chronic stage pathology, we tested transplantation of mouse induced neural stem cells (iNSCs) into the chronically demyelinated corpus callosum (CC) in adult mice. Male C57BL/6 mice fed 0.3% cuprizone for 12 weeks exhibited CC atrophy with chronic demyelination, astrogliosis, and microglial activation. Syngeneic iNSCs were transplanted into the CC after ending cuprizone and perfused for neuropathology 2 weeks later. Magnetic resonance imaging (MRI) sequences for magnetization transfer ratio (MTR), diffusion-weighted imaging (T2), and diffusion tensor imaging (DTI) quantified CC pathology in live mice before and after iNSC transplantation. Each MRI technique detected progressive CC pathology. Mice that received iNSCs had normalized DTI radial diffusivity, and reduced astrogliosis post-imaging. A motor skill task that engages the CC is Miss-step wheel running, which demonstrated functional deficits from cuprizone demyelination. Transplantation of iNSCs resulted in marked recovery of running velocity. Neuropathology after wheel running showed that iNSC grafts significantly increased host oligodendrocytes and proliferating oligodendrocyte progenitors, while modulating axon damage. Transplanted iNSCs differentiated along astrocyte and oligodendrocyte lineages, without myelinating, and many remained neural stem cells. Our findings demonstrate the applicability of neuroimaging and functional assessments for pre-clinical interventional trials during chronic demyelination and detect improved function from iNSC transplantation. Directly reprogramming fibroblasts into iNSCs facilitates the future translation towards exogenous autologous cell therapies.

## Introduction

Recent epidemiological data identified nearly a million adults in the United States alone who are living with a diagnosis of multiple sclerosis (MS), which is almost double prior estimates [1]. This increased appreciation of the MS burden to patients and society emphasizes the urgent need for developing effective treatments. Early in their disease course, relapsing-remitting MS patients often experience periods of autoimmune remission accompanied by spontaneous remyelination [2]. In contrast, the progressive pathology of chronic MS results in sclerotic lesions with axon loss and failed remyelination [3]. Novel treatment strategies are critical to suppress immune attacks, overcome factors limiting remyelination, and prevent neurodegeneration [4–6]. The current studies test a novel neural stem cell-based therapy in an experimental model of chronic demyelination.

Immature oligodendrocyte lineage cells persist in adult human white matter but fail to mature and remyelinate in chronic MS lesions [7–9]. Among interventions to promote remyelination, anti-Lingo-1 and clemastine have advanced to clinical trials in relapsing MS patients [10, 11]. Anti-Lingo-1 and clemastine act by modulating molecular signals that regulate oligodendrocyte differentiation [12–14]. Pre-clinical studies, including those for anti-Lingo-1 and clemastine, have often used acute demyelination models that undergo efficient spontaneous remyelination by endogenous oligodendrocyte progenitor cells (OPCs) [12, 14, 15]. The outcome of these acute stage studies is then based on accelerating remyelination to enhance recovery of function and protect demyelinated axons from further damage. However, acute demyelination models cannot effectively evaluate treatment strategies for chronic demyelination and neurodegeneration [16].

The cuprizone (CPZ) model of corpus callosum (CC) demyelination is well characterized for remyelination studies [17, 18]. CPZ ingestion for 4–6 weeks produces acute demyelination with robust OPC amplification and extensive remyelination [19, 20]. In CPZ and other acute demyelination models, multiple signaling molecules promote remyelination from newly generated oligodendrocytes [2, 21]. Prolonging CPZ ingestion for 10–12 weeks produces chronic demyelination with progressive pathology, including CC atrophy, persistent astrogliosis, OP depletion, and limited remyelination [20, 22–24]. Importantly, CC atrophy correlates with functional outcomes in MS patients [25, 26]. Relevant to modeling sclerotic lesions, chronic CPZ lesions express molecules associated with astrogliosis that inhibit OP differentiation and limit remyelination [23, 27, 28].

The objective of the current study was to employ a combination of translational approaches to evaluate induced neural stem cell (iNSC) transplantation after chronic demyelination. Direct conversion of mouse or human somatic cells to generate iNSCs circumvents the pluripotent stage associated with increased tumorigenesis and genomic instability, with potential as an autologous therapy [29–32]. To model an autologous set-up, we transplanted syngeneic iNSCs into the CC after 12 weeks of CPZ in two cohorts of mice. In one cohort, longitudinal magnetic resonance imaging (MRI) demonstrated the progression of CC pathology throughout the course of CPZ demyelination, and during recovery after iNSC transplantation. In the second cohort, functional testing used Miss-step wheel running to engage the CC and reveal motor deficits after CPZ demyelination [33–35]. Following MRI and behavioral assessments, brain tissue sections were examined for CC atrophy, remyelination, and cellular responses of both transplanted iNSCs and host endogenous cells. Our work provides evidence ofimproved function after iNSC transplantation together with technical advances for translational chronic CPZ model and may pave the way for a novel therapeutic strategy to treat progressive MS by autologous neural stem cell therapies.

## Materials and methods

### Experimental design and statistical analysis

The experiments were designed as pre-clinical trials to test the effect of iNSC transplantation during chronic CPZ demyelination. Experiments were carried out according to the Animal Research Reporting: In Vivo Experiments (ARRIVE) guidelines. Technique validation was performed using pilot cohorts. Two parallel trial experiments each used a non-invasive assessment, MRI (structural) or behavior (functional), as the primary outcome measure. A pre-determined study design was used that stated inclusion/exclusion criteria, randomization procedures, and statistical analyses. All experiments were performed with blinding during implementation and analysis. Sample size estimates were based on prior experiments using each primary outcome measure (MRI or behavior). To reduce variability due to the demyelination produced by CPZ, mice were split into groups balanced by weights and assigned to treatment condition using the Excel RAND command for randomization. After completion of the primary outcome assessment, mice in each experiment were perfused for neuropathology and cell type analyses.

Timelines for the mouse cohorts are shown in the corresponding figures and mouse sample numbers are provided in each legend. A total of 100 mice were used across the experiments in the study. Only male mice were used since CPZ toxicity effects the estrus cycle so that sex cannot be appropriately analyzed as a biological variable [36]. Statistical analysis and graphing was performed using GraphPad Prism software version 8.0 (RRID: SCR_002798). The values are shown as the mean ± standard error of the mean (sem). Statistical significance was determined as *p* < 0.05. The experimental design and statistical analysis for each section is provided below with the technical methods for that study.

### Mice and cuprizone model of demyelination

Mice were housed and cared for in accordance with the National Institutes of Health Guide for the Care and Use of Laboratory Animals. The study protocol was approved by the Institutional Animal Care and Use Committee of the Uniformed Services University of the Health Sciences. Mice were socially housed in 27 cm × 16.5 cm × 12.5 cm cages (2–5 mice per cage) with enrichment objects and maintained on a standard 12 h cycle of daytime light (6:00–18:00). All procedures took place during the daytime light cycle.

C57BL/6 J mice (RRID:IMSR_JAX:000664, Jackson Laboratory, Bar Harbor, MA) at 8 weeks of age were fed pellets containing 0.3% CPZ for 6 weeks or 12 weeks to produce acute or chronic demyelination, respectively. Milled CPZ powder (cat #14690; Sigma-Aldrich; St. Louis, MO) was mixed as 0.3% CPZ in normal chow (diet TD.01453; Harlan Teklad, Frederick, MD) to form pellets that were refreshed every 2–3 days. A dose of 0.3% CPZ in pellets produces CC demyelination approximately equivalent to 0.2% CPZ powder in ground chow [37–39]. Cohorts to be evaluated for remyelination received normal chow pellets for an additional 2 weeks after CPZ removal. Naïve mice continuously fed normal chow pellets served as a control non-demyelinated condition performed simultaneously along with CPZ treated mice. Baseline weights at the start of CPZ or normal chow feeding were similar within each cohort: MRI technical validation cohort (mean ± sem = naïve 25.25 ± 0.47 g; CPZ 25.25 ± 0.43 g), MRI iNSC transplant cohort (CPZ veh 26.24 ± 0.42 g; CPZ iNSC 26.33 ± 0.37 g), wheels iNSC transplant cohort (naïve veh 22.87 ± 0.40 g; CPZ veh 23.76 ± 0.56 g; CPZ iNSC 23.76 ± 0.61 g). Mice remained in good health and regained baseline weights by the end of chronic CPZ administration.

### Magnetic resonance imaging (MRI)

In vivo MRI was performed on a 7 T small animal (20 cm bore) Bruker BioSpec scanner equipped with 12 cm diameter 650 mT/m gradient coils (Bruker BioSpin GmbH, Reinstetten, Germany). MRI with T2 weighting, magnetization transfer ratio (MTR), and diffusion tensor imaging (DTI) was used for longitudinal in vivo analysis of the effects of chronic CPZ ingestion and subsequent iNSC transplantation in the CC. Prior to image acquisition, mice were separated into yoked pairs of similar weight. During each image acquisition, mice were anaesthetized with 1% isoflurane. MRI slices were established using a sagittal localizer so that seven coronal slices were orientated perpendicular to the length of the CC and each scan was aligned with the midline crossing of the anterior commissure positioned in the same coronal slice [40–42].

For DTI [43], a three-dimensional (3D) single-shot echo planar imaging sequence (repetition time/echo time [TR/TE] = 900/36 msec; 1 repetition) was used to acquire 4 unweighted (b = 0 s/mm^2^) and 2 diffusion-weighted images (b = 600, 1200 s/mm^2^) in 14 noncollinear diffusion gradient directions using a Stejskal-Tanner diffusion preparation with parameters of *Δ* = 12 msec and *δ* = 5 msec, field of view (FOV) = 14 × 11.2 mm^2^, matrix = 80 × 64 × 24, slice thickness = 750 μm, 24 slices, voxels = 175 × 175 × 750 μm^3^. A whole brain T2 map was generated using a two-dimensional rapid acquisition with relaxation enhancement (2D RARE, corona [44]) with the following parameters: TR = 4000 msec; TE = 10, 30, 50, 70, 90, 110 msec; RARE factor = 2, number of averages (NA) = 4, FOV = 14 × 12 mm^2^, matrix = 112 × 96 × 18, slice thickness = 750 μm, 18 slices, voxels = 125 × 125 × 750 μm^3^. For use in calculating the magnetization transfer ratio (MTR) [45, 46], 2D RARE sequence was used with TR = 6000 msec, TE = 20 msec, RARE factor = 8, NA = 4, FOV = 14 × 12 mm2, matrix = 112 × 96 × 18, voxels = 125 × 125 × 750 μm^3^, with saturation (*Ms*) and without (*Mo*) using an offset saturation pulse (2 kHz offset 66.7° flip angle). Body temperature was maintained at 36 °C by circulating hot water. Respiration and heart rate were monitored throughout each 2-h imaging session.

T2 and MTR maps along with T2 templates were generated from neuroimaging informatics technology initiative (NIFTI) files and analyzed in either VivoQuant (inviCRO, Boston, MA) (chronic CPZ) or MATLAB (MathWorks, Natick, MA)(chronic CPZ + 2 weeks recovery). The CC ROI was defined as extending from the midline bilaterally to the point of ventral curvature in the external capsule, as in our previous studies (Sullivan et al. 2013 [47]; Yu et al. 2017 [42]). For each mouse, an initial ROI was propagated across images using the T2 templates and the voxel placement checked/corrected manually for each slice. The rostrocaudal extent of the CC was contained within 7 slices. Slices 1 and 7 were omitted to avoid regions of varying fiber directions toward the genu and splenium. Slices 2–6 (approximately 1 mm to − 2.5 mm relative to bregma) were combined as the full CC ROI. DTI data was analyzed with TORTOISE software [48, 49]. Diffusion-weighted imaging artifacts, including motion, eddy currents, and concomitant field distortions, were calculated and combined to enable the image correction to be applied in a single step. Fractional anisotropy (FA), Trace, and axial (AD) and radial (RD) diffusivity maps were computed using a nonlinear tensor estimation with RESTORE option [50]. Diffusion direction-encoded color (DEC) maps were generated from the TORTOISE software to demonstrate fiber orientation. The CC ROIs were overlaid onto the DTI maps for further quantitative analysis.

#### Technical validation for neuroimaging approach

An MRI study with post-imaging neuropathology evaluated the MRI techniques for detection of chronic demyelination at the end of the chronic CPZ period, when iNSCs will be transplanted in the subsequent experiments. This longitudinal design compared naïve mice (*n* = 6) that received normal chow pellets continuously with CPZ treated mice (*n* = 6) at baseline (8 weeks of age), 6 weeks ± CPZ (14 weeks of age), and 12 weeks ± CPZ (20 weeks of age). Two-way ANOVA with repeated measures was used to compare naïve vs. CPZ at each time point, with Bonferroni multiple comparisons test.

#### Neuroimaging analysis of iNSC transplantation effect on CC microstructure

An MRI longitudinal study with post-imaging neuropathology evaluated the effect of iNSC transplantation on CC microstructure during recovery after chronic CPZ. This longitudinal design compared CPZ treated mice injected with vehicle (*n* = 5, one mouse died) or iNSCs (*n* = 6). Scans were performed at baseline (8 weeks of age before starting CPZ), 6 weeks CPZ, 12 weeks CPZ, and then mice were returned to normal chow pellets until the final scan at 14 weeks. Mice received iNSC or vehicle intracerebral injections 1 day after the return to normal chow. Mice were scanned in sets of four per day, which were balanced by weights prior to the start of CPZ. After the week scan at 6 weeks of CPZ, mice within each set of four were randomly assigned, using Excel RAND, to receive iNSC or vehicle injection. The T2 values of the assigned groups were checked to ensure similar CPZ effect within the cohorts at 6 weeks. Two-way ANOVA with repeated measures was used for within subjects comparisons to baseline to evaluate CPZ pathology, and for comparison of each time point for iNSC vs. vehicle, with Bonferroni correction for multiple comparisons.

### Generation and characterization of mouse iNSCs

Mouse C57BL/6 iNSCs that ubiquitously express farnesylated green fluorescent protein (GFP) were generated as previously detailed [29, 51]. Briefly, iNSCs were produced by direct conversion of mouse embryonic fibroblasts through constitutive expression of Sox2, Klf4, c-Myc, and transient expression of Oct4. iNSCs were then transduced with lentivirus containing a farnesylated GFP construct to target GFP to the inner plasma membrane. Prior to characterization and transplantation experiments, iNSCs were cultured as an adherent monolayer, as detailed previously [29, 51].

For in vitro characterization, iNSCs were cultured as either free floating neurospheres in proliferation medium or seeded onto glass coverslips coated with Matrigel™ (Cat# 356234 Corning, Corning, NY) for growth as adherent monolayers in differentiation medium, as previously detailed [51]. Differentiation medium contained NeuroCults basal medium (Cat# 05700, STEMCELL Technologies, Cambridge, MA) with 10% differentiation supplement (Cat# 05703, STEMCELL Technologies) and 100 units penicillin/0.1 mg streptomycin/mL (Cat# 15140122 Invitrogen, Waltham, MA). For immunocytochemistry, neurosphere cultures were fixed on glass coverslips in 4% paraformaldehyde (PFA, Sigma-Aldrich) 2% sucrose in 1X PBS. Neurospheres were incubated with primary antibodies for neural stem/progenitor cell markers Sox2 (rabbit polyclonal, 1:200; Abcam, Cambridge, MA, ab97959, RRID:AB_2341193) and Nestin (chicken polyclonal, 1:200; Abcam, ab134017, RRID:AB_2753197). Differentiation was examined using immunolabeling to detect markers of either the astroglial lineage (glial fibrillary acidic protein, GFAP; rabbit polyclonal, 1:100; DAKO; Carpinteria, CA, Z0334, RRID:AB_10013382), the neuronal lineage (microtubule associated protein-2, MAP-2, rabbit polyclonal, 1:200; Abcam; ab32454, RRID:AB_776174), or the oligodendroglial lineage (O4 mouse monoclonal, 1:10 [52]). PBS with 0.1% Triton-X 100 and 10% normal goat serum for 1 h was used prior to all primary antibodies, but was not used with O4 to prevent Triton-X 100 degradation of the sulfatide epitope [7, 53]. Secondary antibodies used were goat anti-rabbit IgG conjugated with AF647 (1:200; Thermo Fisher, Waltham, MA; A-21245, RRID:AB_2535813) to detect Sox2, and goat anti-chicken conjugated with AF555 (1:200; Thermo Fisher; A-21437, RRID:AB_2535858) to detect Nestin, donkey anti-rabbit IgG F(ab’)2 conjugated with Cy3 (1:400; Jackson ImmunoResearch, West Grove, PA, 711–166-152, RRID:AB_2313568) to detect GFAP, and goat anti-mouse IgM conjugated with Cy3 (1:50; Jackson ImmunoResearch; 115–166-075, RRID:AB_2338707).

#### Technical validation for iNSCs preparations

Frozen iNSCs were thawed and cultured for amplification and characterization for each transplantation experiment cohort. An aliquot of each iNSC preparation used for transplantation was analyzed to confirm neural stem cell characteristics. For differentiation analysis, the number of immunolabeled cells was counted in *n* ≥ 8 non-overlapping fields per sample up to a total of *n* > 300 cells per aliquot. For growth curve analysis, the total number of cells was quantified after each passage of each subculture.

### Cell transplantation into the corpus callosum

For all microinjections into the CC, mice were anaesthetized with isoflurane (induction 3%, maintenance 2%) and a small < 1.0 mm diameter burr hole was drilled into the skull. A Hamilton gas tight syringe (Cat# 7653–01; Hamilton Company, Reno, NV) was used with adapters (Cat# 55750–01; Hamilton Company) and a pulled glass micropipette (outer diameter 50 μm) [54]. Each microinjection targeted the left CC using coordinates (− 1.0 AP, 0.5 ML, − 1.3DV) relative to bregma. An iNSC single cell suspension of 1 × 10^4^ cells in 1 μl of PBS, or PBS as a vehicle control, was injected over a 5 min period.

### Neurologic analysis with the miss-step running wheel assay

Starting the day after iNSC transplantation, and continuing for 2 weeks, mice were singly housed in home cages with a Miss-step running wheel and an optical sensor to detect wheel revolutions (Mouse Miss-step Activity Wheel system #80821, Lafayette Instruments, Lafayette, IN). The Miss-step running wheels have 16 rungs missing from a standard wheel so that the remaining 22 rungs are distributed in an irregular interval pattern [33]. Whiskers were clipped so that the mice learn to locate each rung by bringing the hind paw forward to grasp the same rung as a forepaw [35]. Activity Wheel Monitor software (Lafayette Instruments) counted wheel revolutions at 6 min intervals during the light phase and 1 min intervals during the dark phase. Results were exported to a Microsoft Excel file every 24 h.

#### Technical validation for wheel running effects on oligodendrocyte populations in CC

A study using acute CPZ, which undergoes extensive spontaneous remyelination, tested the effect of the Miss-step wheel exposure on the oligodendrocyte lineage response in the CC. Mice were fed normal chow (naïve, *n* = 6) or CPZ for 6 weeks to produce acute demyelination (acute CPZ, *n* = 12). After 6 weeks, all mice were fed normal chow. The mice were then singly housed in home cages with (naïve, *n* = 6, acute CPZ, *n* = 6), or without (acute CPZ, *n* = 6), Miss-step wheels for 2 weeks. Mice were perfused for in situ hybridization. Across the three conditions, cell densities were quantified from at least 3 sections per mouse and analyzed using one-way ANOVA with Tukey’s multiple comparisons test.

#### Miss-step wheel analysis of iNSC transplantation on CC function

Running on Miss-step wheels, with irregularly spaced rungs, presented a novel motor skill task to evaluate effects of iNSC transplantation on sensorimotor function during recovery after chronic CPZ. Cohorts were maintained under the same conditions for simultaneous automated data collection. The study included four cohorts of 12 mice each, which included naïve with vehicle injection (*n* = 4), CPZ with vehicle injection (*n* = 4), and CPZ with iNSC transplant (*n* = 4). Of the four cohorts run, one cohort was excluded due to the CPZ vehicle condition failing to demonstrate a demyelination associated deficit, compared to naïve. Data from 3 independent cohorts of 12 mice each was combined for analysis of naïve (*n* = 11), CPZ vehicle (*n* = 12), and CPZ iNSC (*n* = 11). Death prior to the experimental endpoint required exclusion of one mouse (CPZ iNSC, *n* = 1). A mouse (naïve, *n* = 1) that did not achieve criterion (average velocity of 10 m/min by day 7) during the learning phase was excluded from analysis of the plateau phase. A value missing due to technical errors of the wheel system was imputed based on the mean (*n* = 1 mouse on 1 d). Velocity measures were compared using repeated measures two-way ANOVA with Sidak’s multiple comparison test. For in situ hybridization analysis in tissues from mice with and without wheel exposure, additional mice (naive veh, *n* = 5; CPZ veh, *n* = 5) were housed singly without wheels in parallel with the wheel mice from two different cohorts.

### Tissue analysis of neuropathology and cellular responses

After completion of imaging or Miss-step wheel assessments, mice were perfused for tissue analysis. Imaging and wheel running cohorts were separately analyzed. Quantification included at least 3 tissue sections per mouse, unless otherwise noted. The number of mice for each condition is provided with each experiment above and is stated in each figure legend. Images for quantification were acquired using an Olympus IX70 fluorescence microscope with a SPOT RT3 camera (Diagnostic Instruments, Sterling Heights, MI). The CC ROI was defined as extending from the midline bilaterally to the point of ventral curvature in the external capsule [42, 47]. Coronal sections within − 0.5 and − 2.0 mm relative to bregma were used for quantification, to focus in the most demyelinated region and minimize variation [40]. Unpaired *t-*tests were used to compare two conditions, i.e. naïve vs. CPZ, or veh vs. iNSCs. After the Miss-step wheel running, further analysis of oligodendrocyte populations for CPZ veh vs. CPZ iNSCs used two-way ANOVA with Sidak’s multiple comparisons test.

#### Immunohistochemistry

Mice were perfused transcardially with 4% PFA, post-fixed overnight at 4 °C, and processed for frozen sectioning. Coronal cryosections (15 μm) were immunostained with primary antibodies to detect either myelin based on myelin oligodendrocyte glycoprotein (MOG, mouse monoclonal, 1:100; EMD Millipore, Burlington, MA; mab5680, RRID:AB_1587278), astrocytes with GFAP (rabbit polyclonal, 1:500; DAKO; Z0334, RRID:AB_10013382), microglia/macrophage with IBA1 (rabbit polyclonal, 1:500; Wako, Richmond, VA; 019–19,741, RRID:AB_839504), oligodendrocyte lineage cells with Olig2 (rabbit polyclonal, 1:500; EMD Millipore, ab9610, RRID:AB_570666), OPCs with NG2 (rabbit polyclonal to ectodomain; generous gift from William Stallcup; RRID:AB_2572306) and neural stem/progenitor cells with Sox2 (rabbit polyclonal, 1:200; Abcam, ab97959, RRID:AB_2341193). Cells undergoing active proliferation were identified by Ki67 (rabbit polyclonal, 1:500; Abcam ab 15,580, RRID:AB_443209). Axons were identified by neurofilament heavy chain protein (NF-H) rabbit polyclonal antibody (1:500; RPCA-NF-H Encor Biotechnology, Gainesville, FL, RRID:AB_2572360) with the proportion with axon damage immunolabeled for SMI32 mouse IgG1 monoclonal antibody, which recognizes nonphosphorylated neurofilaments (1:1000; Sternberger monoclonal SMI32; 801,701 Biolegend, San Diego, CA, RRID:AB_2564642). The secondary antibodies used were donkey anti-rabbit IgG F(ab’)2 (Jackson ImmunoResearch, West Grove, PA) conjugated with Cy3 (1:400 or 1:500; 711–166-152, RRID:AB_2313568) or AF647 (1:100; 711–606-152, RRID:AB_2340625), donkey anti-mouse IgG IgG F(ab’)2 conjugated with Cy3 (1:100; Jackson ImmunoResearch; 715–166-150, RRID:AB_2340816), goat anti-rabbit IgG conjugated with AF647 (1:200; Thermo Fisher; A21245, RRID:AB_2535813) and goat anti-mouse IgG conjugated with AF555 (1:500; Thermo Fisher; A21422, RRID:AB_2535844). Nuclei in all sections were counterstained with DAPI (Sigma-Aldrich). The GFP expression of iNSCs was sufficient for direct detection without using additional antibody detection.

#### In situ hybridization

In situ hybridization using a proteolipid protein (*Plp1*) riboprobe was performed as previously described [55, 56]. The Enpp6 plasmid vector (pCMV-SPORT6, gift from Dr. William D. Richardson, University College London) was used to generate the *Enpp6* riboprobe (Xiao et al. 2016 [57]). In 15 μm coronal cryosections, hybridized *Plp1* or *Enpp6* riboprobe was detected with alkaline phosphatase-conjugated sheep anti-digoxigenin antibody and incubation in substrate solution (nitroblue tetrazolium chloride/5–bromo-4–chloro-3–indolyl-phosphate [NBT/BCIP]; Dako).

#### Quantification details for CC area, myelin, astrogliosis and microglia activation

Immunolabeling within the CC ROI was quantified on images acquired with a 10x objective. Metamorph software (RRID:SCR_002368; Molecular Devices, Downington, PA) was used to measure the total CC ROI area in coronal sections immunolabeled for MOG along with DAPI staining of nuclei for cytoarchitecture of CC as distinct from adjacent regions. Myelination of the CC was measured based on pixel intensity values to determine the MOG immunolabeled pixels above background levels using the Metamorph thresholding function [20]. Similar thresholding was used to quantify astrogliosis and microglia activation based on GFAP and IBA1 immunoreactivity, respectively.

#### Quantification details for structure tensor analysis of astrocytes and myelin

NIH ImageJ software (ImageJ, RRID:SCR_003070) with the OrientationJ Plug-in (RRID:SCR_014796, http://bigwww.epfl.ch/demo/orientation/) was used for structure tensor analysis [58]. Images were acquired with a 10x objective. Using the polygon tool, the ROI was selected within the CC under the medial extension of the cingulum. This CC region avoids the curvature toward the midline and the crossing fibers that are present more laterally. The program computes the microscopic, or local, orientation and local coherence for each pixel. The local orientation uses a color map to represent the directional distribution. The local coherence is a measure of the alignment of anisotropic domain tensors. Both the anisotropy of a local domain and the coherence of domains within a voxel contribute to fractional anisotropy [59].

#### Quantification details for oligodendrocyte lineage populations

Oligodendrocyte counts in the CC were based on in situ hybridization and quantified from bright field images with the CC ROI area measured using Spot Advanced Software (RRID: SCR_014313; Spot imaging solutions, Sterling Heights, MI). *Plp1*^*low*^ expressing cells had mRNA transcripts localized mainly in the perinuclear cytoplasm; in *Plp1*^*high*^ expressing cells, darker substrate reaction was evident in the cell body and extended out into processes [20, 47, 60]. Only cells with strong substrate reaction for *Enpp6* transcript levels were counted as specific labeling of newly formed oligodendrocytes [57]. Quantification of proliferating OPCs in the CC and cingulum were identified based on Ki67 immunoreactive nuclei and NG2 immunolabeling of the cell body and processes. Ki67 and NG2 analysis included only one section per mouse due to the limited availability of tissue within the defined coronal levels.

#### Quantification details for axon damage

Confocal images were acquired at 63x and quantified in maximum intensity projections of the ROI (59.70 μm, y: 59.70 μm, z: 1.60 μm) in the cingulum. The ROI was positioned adjacent to the CC and centered under the peak of the cingulum. Individual axons were manually counted as immunolabeled for NF-H with or without co-labeling for SMI32. Nuclei were counted simultaneously. Ipsilateral and contralateral sides were quantified in at least 3 sections per mouse.

### Transplanted iNSC localization and differentiation in vivo

Transplanted iNSCs were quantified by direct visualization of GFP expression using a 40x objective on an Olympus IX-70 microscope. Tissue sections were analyzed from mice in the imaging (*n* = 6) and Miss-step wheel assessments (*n* = 11) that were used for quantification of MOG, GFAP, and IBA1 immunoreactivity. The in situ hybridization reaction for *Plp1* precluded identification of GFP expression from iNSCs. Additional tissue sections were immunostained for labeling of iNSCs with cell type markers. Overall, this iNSC cell type quantification included at least 6 mice per cell type immunostain with at least 3 sections analyzed per mouse combining to approximately 200 iNSCs each for Sox2 and for Olig2, with approximately 600 iNSCs counted for GFAP which included sections from the neuropathology analysis of astrogliosis.

Transplanted iNSCs were analyzed in vivo only within coronal sections from rostrocaudal levels matching the neuropathology ROI (− 0.5 mm to − 2.0 mm from bregma), which focused on the most demyelinated CC region and minimize variation [40]. Regions of the cerebral cortex and cingulum above the CC, and hippocampus below the CC, were quantified within the same coronal sections as the CC. In total, 1585 iNSC cells were counted from among mice combined from MRI and Miss-step wheel cohorts and graphed to represent the distribution within the tissue as white matter (CC, cingulum) and adjacent gray matter (cortex, hippocampus). Contingency tables were used to analyze in vivo differentiation of iNSCs after transplantation with Fisher’s exact test for statistical significance.

Additional images of GFP cells were acquired on a Zeiss 700 laser scanning confocal microscope (Carl Zeiss, San Diego, California; RRID:SCR_017377) with individual laser lines sequentially collected for each channel using a Plan-Apochromat 63x/1.4 oil objective. An optical image stack was acquired, and maximum intensity projection images were generated from each image stack with Zen software (Carl Zeiss, ZEN Digital Imaging for Light Microscopy, RRID:SCR_013672).

## Results

### Characterization of iNSC preparations used for transplantation

**Fig. 1:**
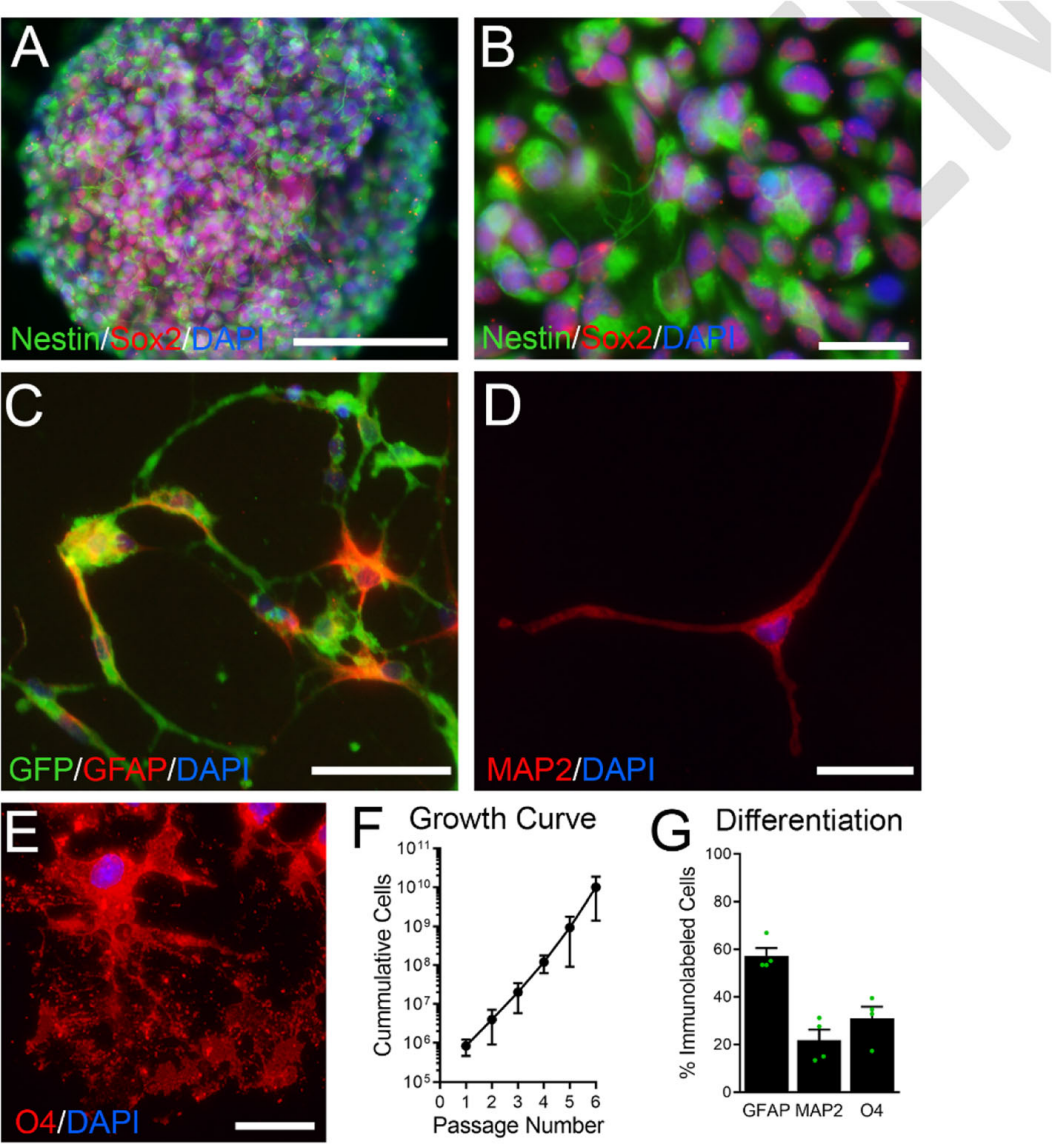
Mouse induced neural stem cells (iNSCs) exhibit stem cell characteristics in vitro. **a-b:** In vitro, iNSCs form neurospheres (**a**) and express NSC markers Sox2 (red) and Nestin (pseudocolored green). **c-e:** Following differentiation of iNSCs, constitutively expressed green fluorescent protein (GFP) is visible in all cells (**c**) while cells differentiated along an astrocytic lineage exhibit immunolabeling for GFAP (red, **c**). In addition, differentiation along neuronal (MAP2; red, **d**) and oligodendroglial (O4; red, **e**) lineages shows multipotency of iNSCs (GFP not shown). **f:** In non-differentiation conditions, iNSCs exhibit an exponential proliferation rate expected for stem cell growth based on quantification of each subculture after each passage. **g:** Quantification of iNSC multipotent differentiation from separately revived vials (*n* = 4). Data are mean values ± sem. Scale bar A = 100 μm, B, D, E = 20 μm, C = 40 μm

Induced neural stem cells (iNSCs) have the potential to self-renew and to differentiate along three neural lineages [29, 31, 32, 51]. Following revival and expansion, iNSCs were assessed for self-renewal as neurospheres (Fig. 1a-b) and differentiation along neural lineages (Fig. 1c-e). In vitro, iNSCs demonstrated immunolabeling for neural stem/progenitor cell markers, Nestin and Sox2, and neurosphere formation (Fig. 1a-b). iNSC self-renewal was exhibited by log phase cell amplification after each passage (Fig. 1f). Following growth in differentiation media, iNSCs acquired markers for either astroglial (GFAP, Fig. 1c), neuronal (MAP2, Fig. 1d), or oligodendroglial (O4, Fig. 1e) lineages. Differentiation was confirmed for each aliquot of iNSCs used for transplantation experiments (Fig. 1g).

### Longitudinal MRI technique evaluation with post-imaging validation of chronic CPZ pathology

**Fig. 2:**
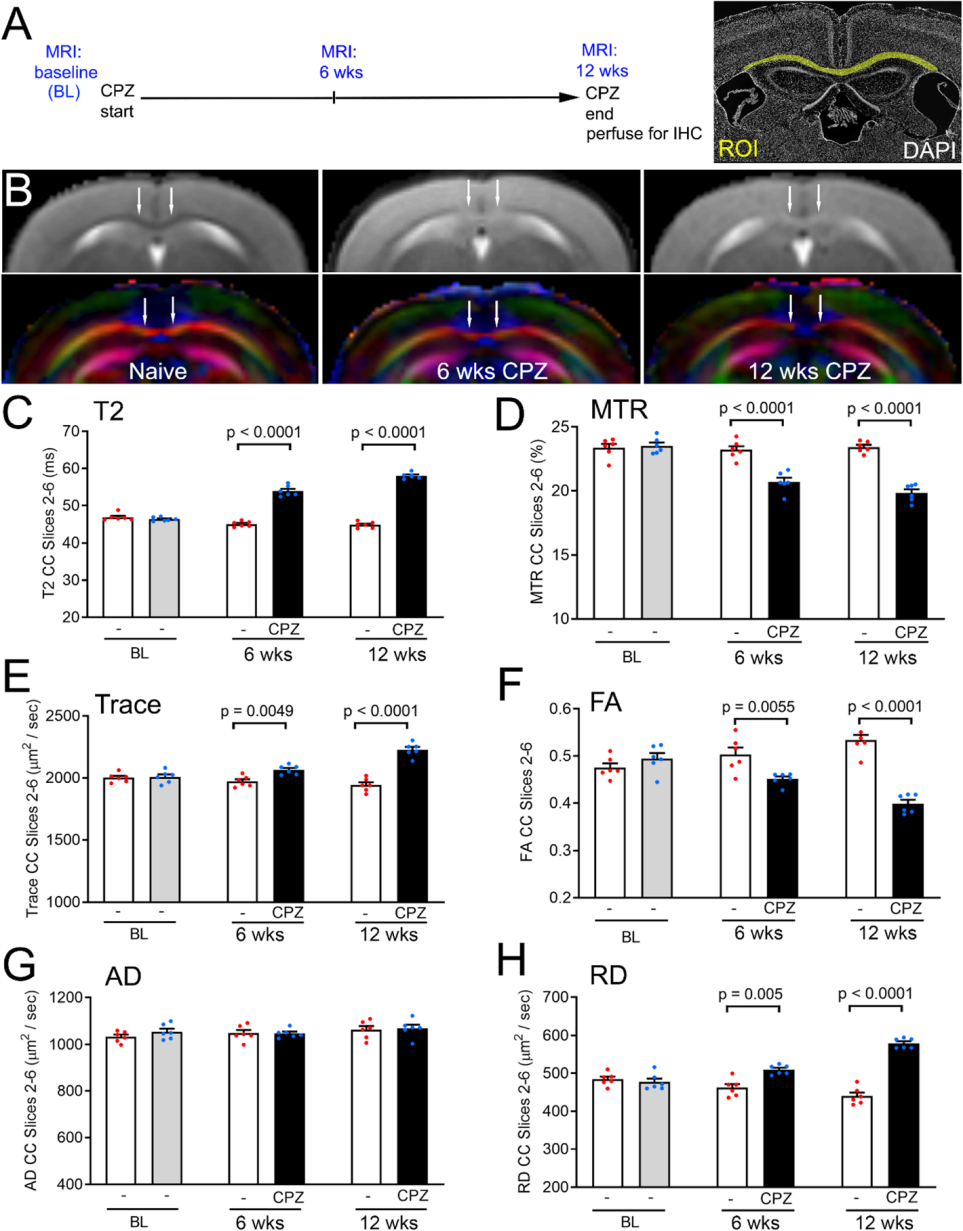
MRI techniques detect progression of corpus callosum (CC) pathology during acute through chronic cuprizone (CPZ). **a:** Experimental timeline for longitudinal in vivo magnetic resonance imaging (MRI) of the same mice scanned three times: at baseline (BL, 8 weeks of age prior to the start of CPZ), again after acute CPZ (6 weeks) and finally after chronic CPZ (12 weeks). Naïve mice were scanned at matched time points but continuously fed normal chow pellets without CPZ. CC region-of-interest (ROI) for quantification shown in yellow with DAPI nuclear staining of cytoarchitecture. **b:** Representative T2-weighted images with corresponding direction encoded (DEC) color images below for each set. T2 panels show increased signal intensity in the CC during acute and chronic CPZ while DEC maps show altered CC microstructure, as compared to naïve mice (age-matched to the 12 week CPZ time point). Arrows indicate medial CC regions that show CPZ effect. Colors represent fiber directions as red (medial-lateral), blue (anterior-posterior), and green (superior-inferior). **c-h:** Quantitative analysis of the CC shows significant differences between naïve and CPZ mice at both 6 weeks and 12 weeks after the start of CPZ using values from T2-weighted imaging (**c**), magnetization transfer ratio (MTR, **d**), and diffusion tensor imaging parameters of trace (**e**), fractional anisotropy (FA; **f**), and radial diffusivity (RD; **h**). Axial diffusivity (AD, **g**) remained unchanged. Bar color reflects the treatment condition. The naïve cohort (white; no cuprizone shown as “-“under bars) was scanned along with the CPZ cohort at each of the three time points. The CPZ cohort was scanned at baseline (gray; “-“before starting CPZ) and during demyelination (black; at 6 and 12 weeks of CPZ). Data are mean values ± sem. Two-way ANOVA with repeated measures and Bonferroni multiple comparisons test for naïve (*n* = 6) versus CPZ (*n* = 6) mice

Prior to studies of iNSC transplantation, we first tested the set of MRI techniques for quantification of the progression of CPZ acute and chronic demyelination (Fig. 2). These techniques quantitatively demonstrate MRI changes in the CC at 6 and 12 weeks after CPZ in comparison with naïve mice fed normal chow across the same time course (Fig. 2a). The T2 and direction-encoded color images show that acute and chronic CPZ changes are most marked in the medial CC (Fig. 2b). CPZ increased the T2-weighted signal, which reflects increased unbound water, while reducing the magnetization transfer ratio (MTR) associated with water bound to membranes, including myelin (Fig. 2c-d). Diffusion tensor imaging (DTI) values provided complementary measures (Fig. 2e-h). Trace values indicated increased water diffusion within the CC after CPZ. Reduced fractional anisotropy (FA) was driven by increased radial diffusivity (RD), which can be associated with demyelination. Axial diffusivity (AD) was not altered after 6 or 12 weeks of CPZ, which is in agreement with our prior studies that found axonal beading and altered AD at 3–4 weeks of CPZ was attenuated by the 6 week time point [40, 41, 61, 62].

**Fig. 3:**
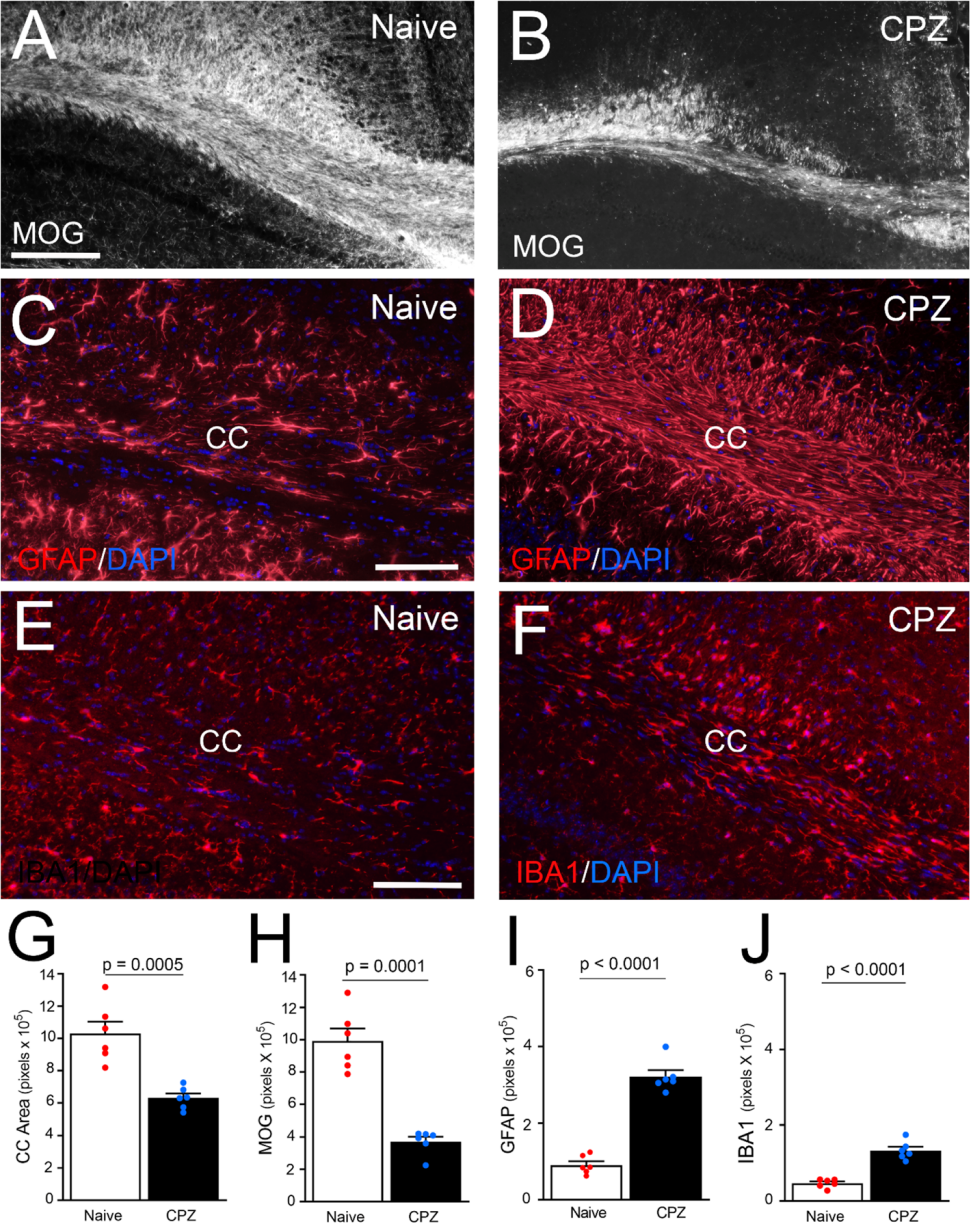
Post-MRI pathology after chronic CPZ. **a-f:** Coronal sections from naïve and 12 wk. CPZ perfused after final MRI (Fig. 2) and immunolabeled for either myelin (MOG; **a**-**b**), astrocytes (GFAP; **c**-**d**), or microglia (IBA1; **e**-**f**), along with DAPI nuclear stain (blue; **c**-**f**). **g-j:** Quantification of immunolabeling in this post-MRI cohort shows that chronic CPZ results in significant CC atrophy (**g**) with demyelination (**h**), astrogliosis (**i**), and microglial activation (**j**). CC area was measured using DAPI nuclear staining of cytoarchitecture and MOG immunoreactivity. Bar color reflects condition as naïve (white; no cuprizone), or demyelinated (black; 12 weeks of cuprizone). Data are mean values ± sem. Naïve (*n* = 6) compared to chronic CPZ (*n* = 6) using *t*-tests. Scale bars A, B: shown in A = 200 μm; C, D: shown in C = 100 μm; E, F: shown in E = 100 μm

Post-imaging neuropathology focused on CC myelination and neuroinflammation to characterize the chronic lesion environment into which iNSCs would be transplanted in subsequent experiments (Fig. 3). Mice from the MRI cohort were perfused after the final scan. Chronic CPZ mice had significant CC atrophy, based on the reduced area measured, and significant demyelination demonstrated with immunolabeling of myelin. Chronic CPZ also resulted in persistent astrogliosis, which is a feature of sclerotic lesions. Increased microglial signal also indicated CC neuroinflammation.

### Longitudinal MRI and post-imaging neuropathology changes after iNSC transplantation during chronic demyelination

**Fig. 4:**
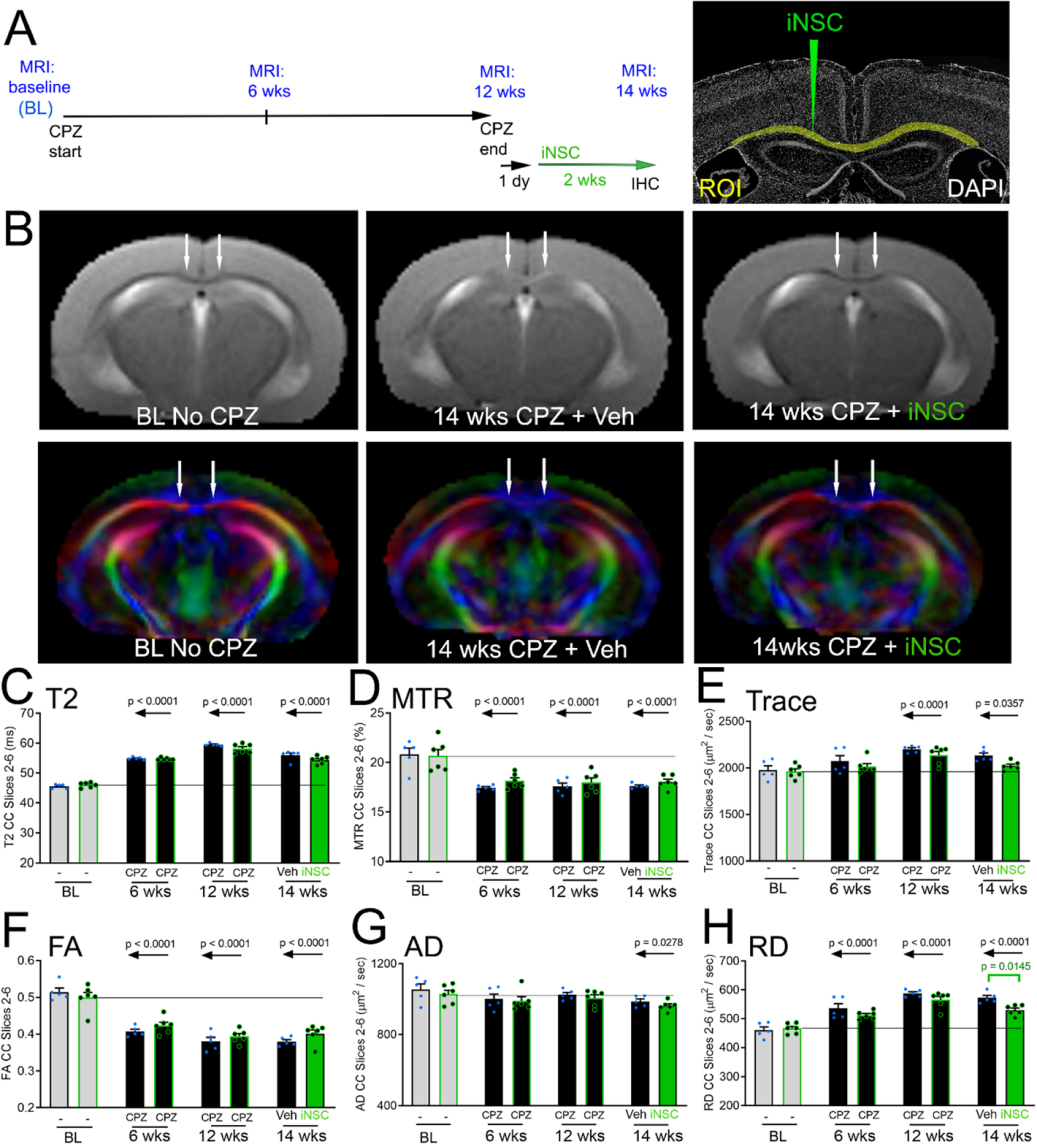
MRI radial diffusivity indicates reduced CC pathology with iNSC transplantation. **a:** Experimental timeline for longitudinal imaging of mice across 4 time points: baseline (BL) before initiating CPZ, at 6 weeks of CPZ, and at 12 weeks of CPZ before and finally at 2 weeks after iNSC, or vehicle, injection. After the scan at 6 weeks of CPZ, mice were randomized to vehicle or iNSC injection. After 12 weeks of CPZ, mice were returned to normal chow pellets for 1 day before intracerebral injection. CC region-of-interest (ROI) for quantification shown in yellow with iNSC injection path shown in green, with DAPI nuclear staining of cytoarchitecture. **b:** Representative T2-weighted and DEC images. Arrows indicate medial CC regions that show CPZ effect. Colors reflect fiber directions as red (medial-lateral), blue (anterior-posterior), and green (superior-inferior). **c-h:** Quantitative analysis of the CC shows significant differences from baseline result from CPZ administration using values from T2-weighted imaging (**c**), MTR (**d**), trace (**e**), FA (**f**), and RD (**h**). The AD values only differ from baseline at the 14 week time point (**g**). The comparison of iNSC transplantation versus vehicle (Veh) shows a significant effect in RD values (**h**, green bracket). Horizontal line shown extends from the baseline value of the cohort that later received iNSCs after 12 weeks of CPZ. Bar color reflects condition as baseline (gray fill), demyelinated with CPZ (black fill) or iNSC transplant after chronic CPZ (green fill). Borders around bars denote same mice that received iNSC (green border) or vehicle (black border) after ending cuprizone. Data are mean values ± sem. Two-way ANOVA with repeated measures and Bonferroni multiple comparisons test for CPZ with injection of vehicle (*n* = 5) versus iNSCs (*n* = 6)

Longitudinal MRI scans of each mouse at multiple time points were used to demonstrate the progression of CPZ pathology before, and then after, iNSC or vehicle injection (Fig. 4a). Representative T2 and direction-encoded color images illustrate baseline, i.e. before initiating CPZ, for comparison with the final scan after CPZ and either vehicle or iNSC injection (Fig. 4b). Robust reproducibility is indicated by the similar changes observed from baseline to 6 and 12 week CPZ time points in this cohort (Fig. 4c-h) relative to naïve mice and baseline vs. 6 and 12 week CPZ time points in the validation cohort (Fig. 2c-h). At 12 weeks of CPZ, values are increased for T2, Trace and RD; CPZ decreased MTR and FA, while AD was not changed at 12 weeks (Fig. 4c-h). After removal of CPZ and return to normal chow, mice received iNSC or vehicle injection into the CC. Notably, the iNSC transplants resulted in significant recovery of DTI RD values (Fig. 4h), suggesting an iNSC effect on CC microstrucuture that did not significantly change the other imaging parameters (Fig. 4c-g).

**Fig. 5:**
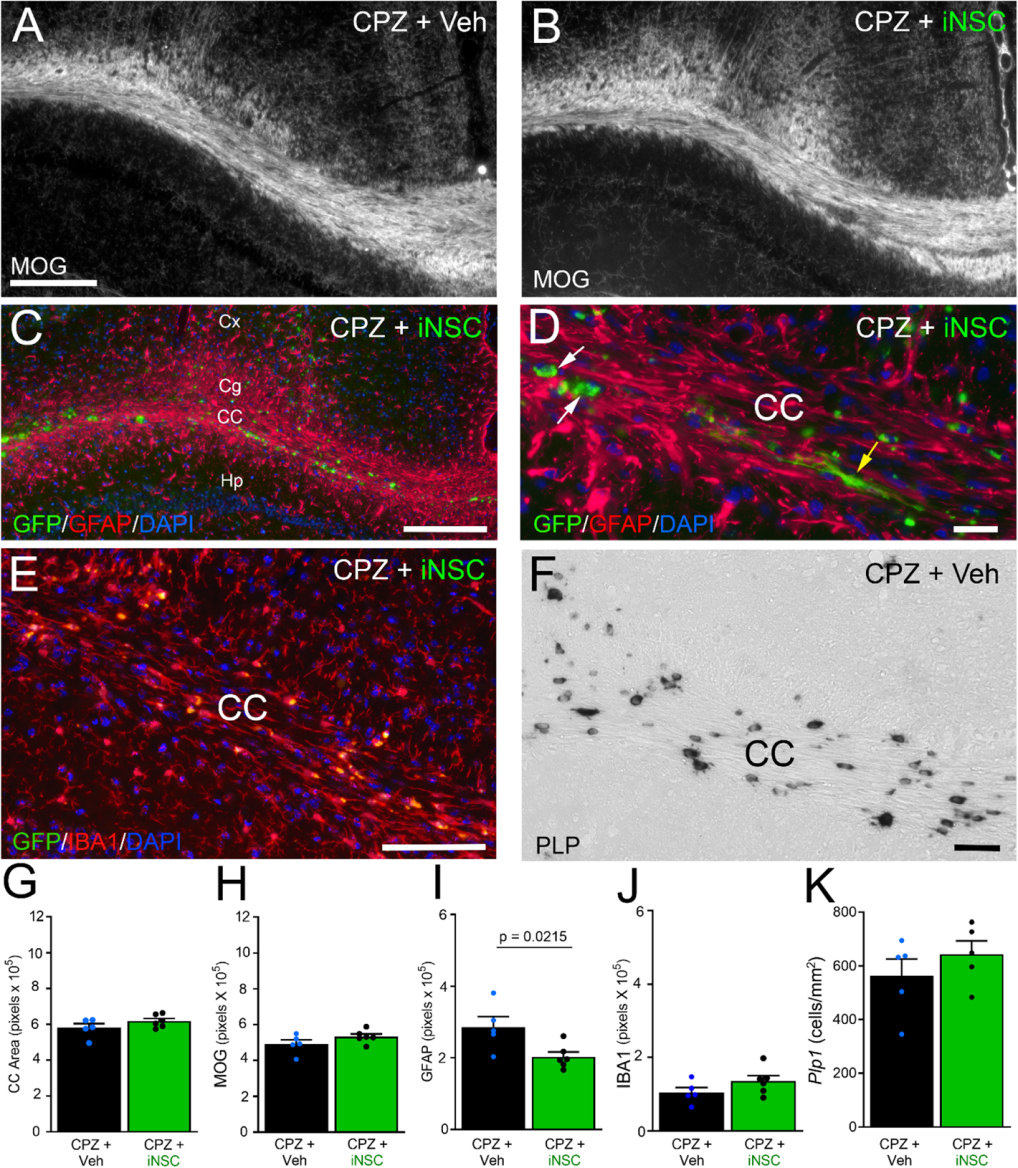
Post-MRI pathology shows iNSC reduction of astrogliosis. **a-e:** Coronal sections from chronic CPZ mice with vehicle (Veh) or iNSC injection immunolabeled to detect myelin (MOG; **a**-**b**), or the innate immune response of astrocytes (GFAP; **c**-**d**) or microglia (IBA1; **e**) with DAPI nuclear stain (blue; **c**-**e**). Panel C shows an example of transplanted iNSCs (green, GFP) in the CC and adjacent white matter (cingulum; Cg) and gray matter regions (cortex, Cx; hippocampus, Hp). Panel D shows higher magnification of iNSCs (green, GFP) in the CC with examples of round cells (white arrows) and elongated cells with extended processes (yellow arrow). Panel E shows IBA1 immunolabeling of microglia (red) with lipofuscin granules (yellow autofluorescence) that accumulate after chronic CPZ. **f:** Coronal CC sections processed for in situ hybridization to detect oligodendrocytes expressing proteolipid protein (*Plp1*). **g-k:** Quantification of tissue analysis in this post-MRI cohort shows CC atrophy and demyelination are not attenuated by iNSCs (**g**, **h**). Transplanted iNSCs reduced astrogliosis (**i**), but did not change the microglial (**j**) or oligodendrocyte response (**k**). Bar color reflects condition as chronic cuprizone followed by vehicle injection (black) or iNSC transplant (green). Data are mean values ± sem. Vehicle (*n* = 5) and iNSC (*n* = 6 IHC, *n* = 5 *Plp1*) conditions were compared using *t*-tests. Scale bars: A, B shown in A = 200 μm; C = 200 μm; D = 20 μm; E = 100 μm; F = 40 μm

Post-imaging neuropathology focused on CC myelination and neuroinflammation, with additional analysis of the endogenous oligodendrocyte population after iNSC transplantation (Fig. 5). Mice that received iNSCs did not exhibit overt changes in CC myelination, as compared to vehicle-injected mice (Fig. 5a-b). Transplanted iNSCs were found in the CC and the overlying white matter of the cingulum, and in adjacent gray matter of the adjacent cortex and hippocampus (Fig. 5c). The iNSCs exhibited mainly rounded morphologies, while some cells had extended processes (Fig. 5d). To determine whether transplanted iNSCs elicited a response among endogenous CC cells, sections were examined using immunolabeling for GFAP for astrocytes (Fig. 5c-d), IBA1 for microglia (Fig. 5e), and in situ hybridization to detect oligodendrocyte expression of *Plp1* (Fig. 5f). The findings of CC atrophy and demyelination at 12 weeks of CPZ (Fig. 3g-h) persisted at similar levels after the 2-week recovery period on normal chow, and were not attenuated by iNSC transplantation (Fig. 5g-h). Interestingly, astrogliosis persisted in vehicle-injected mice and was significantly reduced with iNSC transplantation (Fig. 5i). The microglial immunoreactivity did not show an effect of iNSC transplantation (Fig. 5j). The oligodendrocyte population appeared only slightly increased after iNSC transplantation, and did not reach a statistically significant effect (Fig. 5k).

**Fig. 6:**
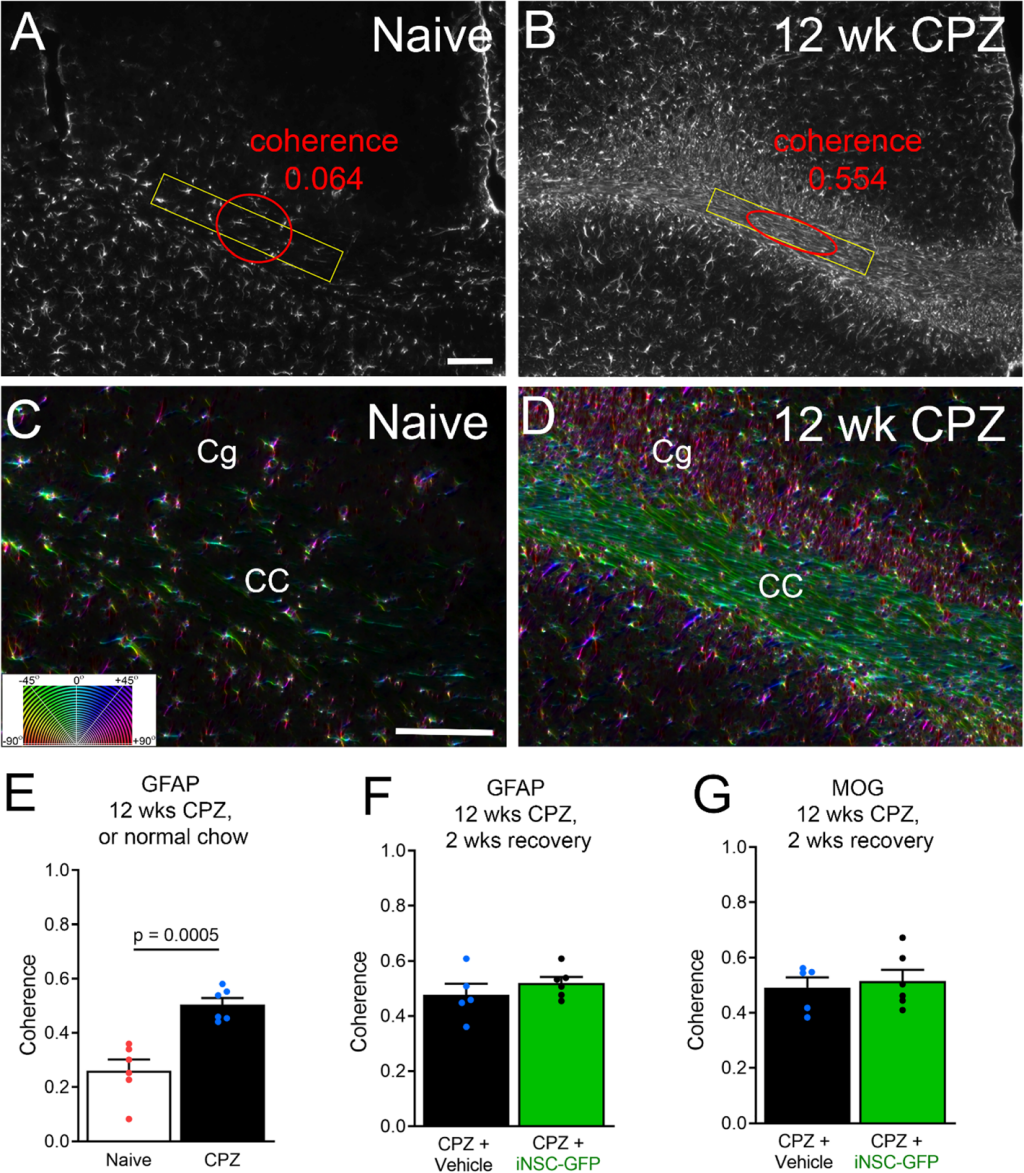
Astrogliosis of chronic CPZ increases coherence within CC. **a-b:** Coronal sections from naïve and 12 week CPZ mice perfused after final MRI and immunolabeled for GFAP to detect astrocytes. Coherence of astrocyte morphology was measured in a CC area (yellow rectangle) of relatively aligned anatomical structure. In the non-pathological CC of naïve mice, the astrocyte density is low and cell processes extend in many directions, resulting in close to isotropic coherence (**a**; red). After CPZ, astrocytes have a high density and align parallel to axons in the chronically demyelinated CC, so that the GFAP immunoreactivity exhibits anisotropic coherence (**b**, red). **c-d:** Color maps illustrating the orientation (**c**, inset) of the astrocyte cell bodies and processes in higher magnification images taken from the same sections as in A and B. In naïve mice, astrocyte cell bodies and processes exhibit diverse color encoding indicating orientation in many directions (**c**). After CPZ, astrocytes are aligned parallel to axons in the CC (green color orientation) and in the perpendicularly aligned cingulum (Cg) tract (magenta color orientation). **e-g:** Quantification of the coherence shows a significant increase in CPZ demyelinated mice (**e**) that does not recover during the 2 weeks on normal chow (**f**), and is not changed in iNSC transplant mice (**f**). In sections immunolabeled for MOG (**g**), the coherence for myelin is similar to that measured for astrocytes (**f**-**g**). Bar color reflects condition as chronic cuprizone followed by vehicle injection (black) or iNSC transplant (green). Data are mean values ± sem. Mouse numbers shown in Figs. 3 and 5, with comparison by *t*-test. Scale bars A, B shown in A = 100 μm; C, D shown in C = 100 μm

The astrogliosis is a remarkable feature of this chronic demyelination model (Figs. 3c-d, 5c-d). Given the high astrocyte density and aligned orientation, we examined the structure tensor characteristics of CC immunolabeled astrocytes after chronic CPZ (Fig. 6). Structure tensor analysis applied to histological tissue can quantify microscopic features that add to the interpretation of tissue microstructure, and has been used for analysis of myelin immunolabeling in chronic CPZ [58, 59, 63]. The images used to quantify GFAP immunoreactivity post-MRI (Figs. 3i, 5i) were used for structure tensor analysis. In naïve mice, GFAP immunolabeled cells had a relatively isotropic appearance (Fig. 6a). After chronic CPZ, the GFAP immunolabeled cells exhibited coherence of local domains for effective anisotropy (Fig. 6b). In naïve mice, astrocyte processes extended in many directions, as indicated by varied color encoding of the orientation (Fig. 6c). After CPZ, reactive astrocytes were highly oriented in parallel to the axis of axons, as indicated by the mainly green encoded orientation in the CC but magenta in the perpendicularly oriented cingulum tract (Fig. 6d). Microscopic, or local, GFAP coherence was significantly increased within the CC after 12 weeks of CPZ compared to naïve mice (Fig. 6e). Surprisingly, the GFAP coherence level was as high as that measured for MOG immunolabeling of myelin (Fig. 6f, g). Myelin and axons are considered major components influencing diffusion tensor imaging values, although cell density and edema are also important factors [64]. These results raise the possibility that astrogliosis can significantly contribute to microstructure signal, which may be relevant for sclerotic MS lesions. Reduced overall GFAP immunolabeling after iNSC transplantation (Fig. 5i) may reduce the overall anisotropic contribution of reactive astrocytes among all the cellular structures contributing to the DTI FA. The DTI AD component of FA is strongly driven by axons [61], and was not significantly different after iNSC transplantation (Fig. 4g). Conversely, the DTI RD component can be driven by myelin changes [61]. Our data suggest that the reduced DTI RD after iNSC transplantation (Fig. 4h) may possibly be influenced by changes in reactive astrogliosis after chronic CPZ, since GFAP and MOG immunoreactivity exhibited similar coherence (Fig. 6f-g). However, our 2-dimensional pixel scale structure tensor analysis can reveal local microstructure features but cannot demonstrate specific correlation to the much larger 3-dimensional DTI voxel scale features.

### Reduced neurologic impairment after iNSC transplantation during chronic demyelination

**Fig. 7:**
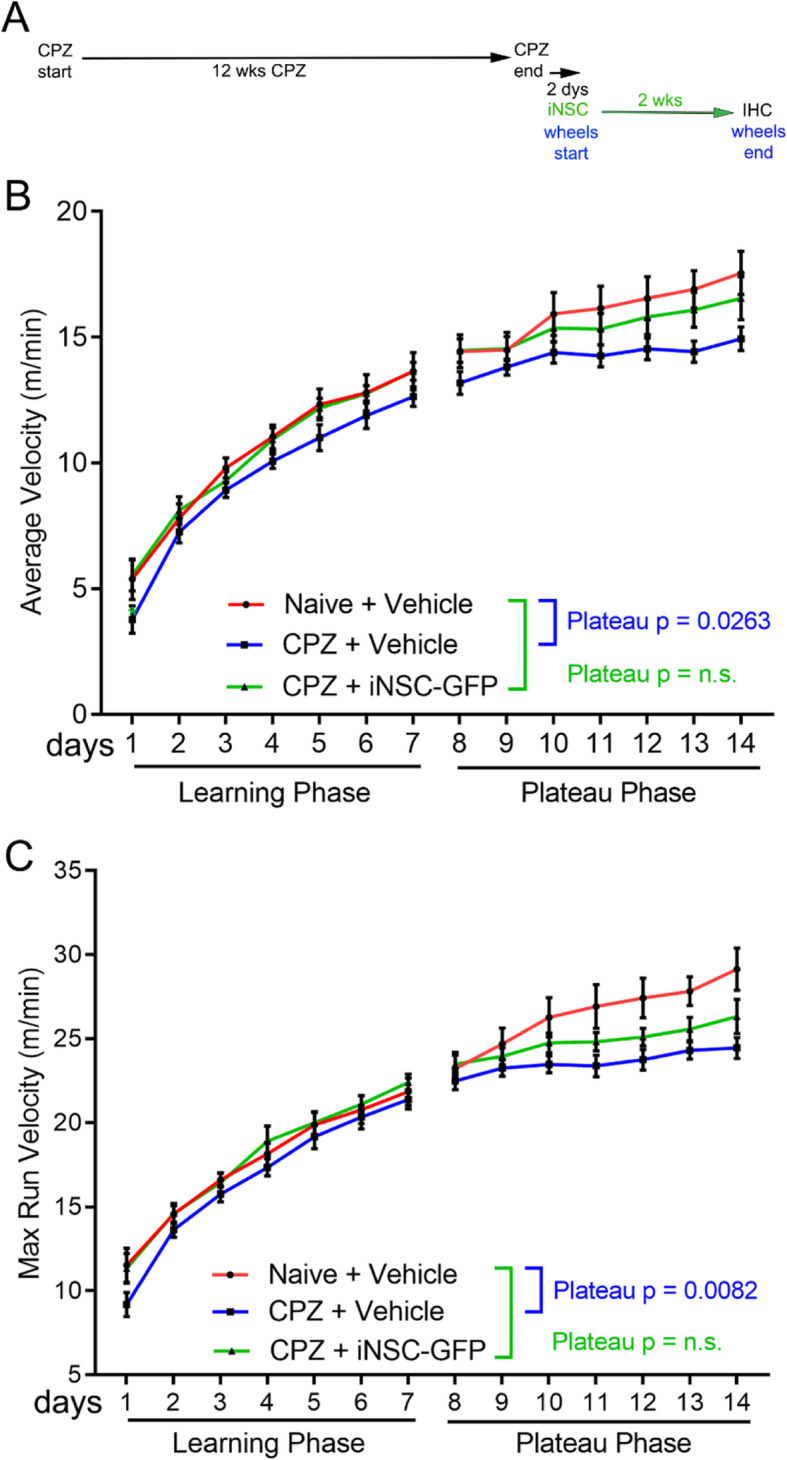
Recovery of motor function with iNSC transplantation. **a:** Experimental timeline for functional assessment after iNSC (or vehicle) injection. After 12 weeks of CPZ, mice were returned to normal chow with intracerebral injection performed the following day and exposure to Miss-step wheels the next day. Miss-step wheels with irregularly spaced rungs present a motor skill task mice must learn to run rapidly (week 1); the subsequent plateau velocity reveals deficits of bilateral sensorimotor function (week 2). **b-c:** The average velocities (**b**) and the maximum velocities (**c**) show deficits in vehicle-injected CPZ mice during the learning phase that reach statistical significance during the plateau phase, as compared to naïve mice. CPZ mice injected with iNSCs show recovery in that both average and maximum velocities are not significantly different from the naïve mice. Velocity measures were compared using two-way ANOVA with Sidak’s multiple comparison test. Data are mean values ± sem. Data from 3 independent cohorts was combined for analysis of naïve (*n* = 11), CPZ vehicle (*n* = 12), and CPZ iNSC (*n* = 11)

We next tested whether iNSC transplantation could reduce neurologic functional deficits after chronic CPZ (Fig. 7). Running on Miss-step wheels, with irregularly spaced rungs, was used because this assessment is sensitive to deficits associated with CPZ demyelination and targeted to CC function [33, 57]. After 12 weeks of CPZ, mice were returned to normal chow and then received iNSC or vehicle injections followed by exposure to Miss-step wheels for 2 weeks (Fig. 7a). Age matched naïve mice received vehicle injections and were maintained within the CPZ cohorts. Comparison of vehicle-injected controls showed significant deficits between naïve and chronic CPZ, which were evident in both the average daily running velocity (Fig. 7b) and the maximal daily running velocity (Fig. 7c). Chronic CPZ impaired motor skill learning (learning phase) with significant performance deficits (plateau phase). On the contrary, mice with iNSC transplantation did not have significant deficits in either the average or maximum running velocities after chronic demyelination, compared to naïve mice (Fig. 7b-c), suggesting improved motor learning and performance.

### Increased oligodendrocyte response to iNSC transplantation during chronic demyelination

**Fig. 8:**
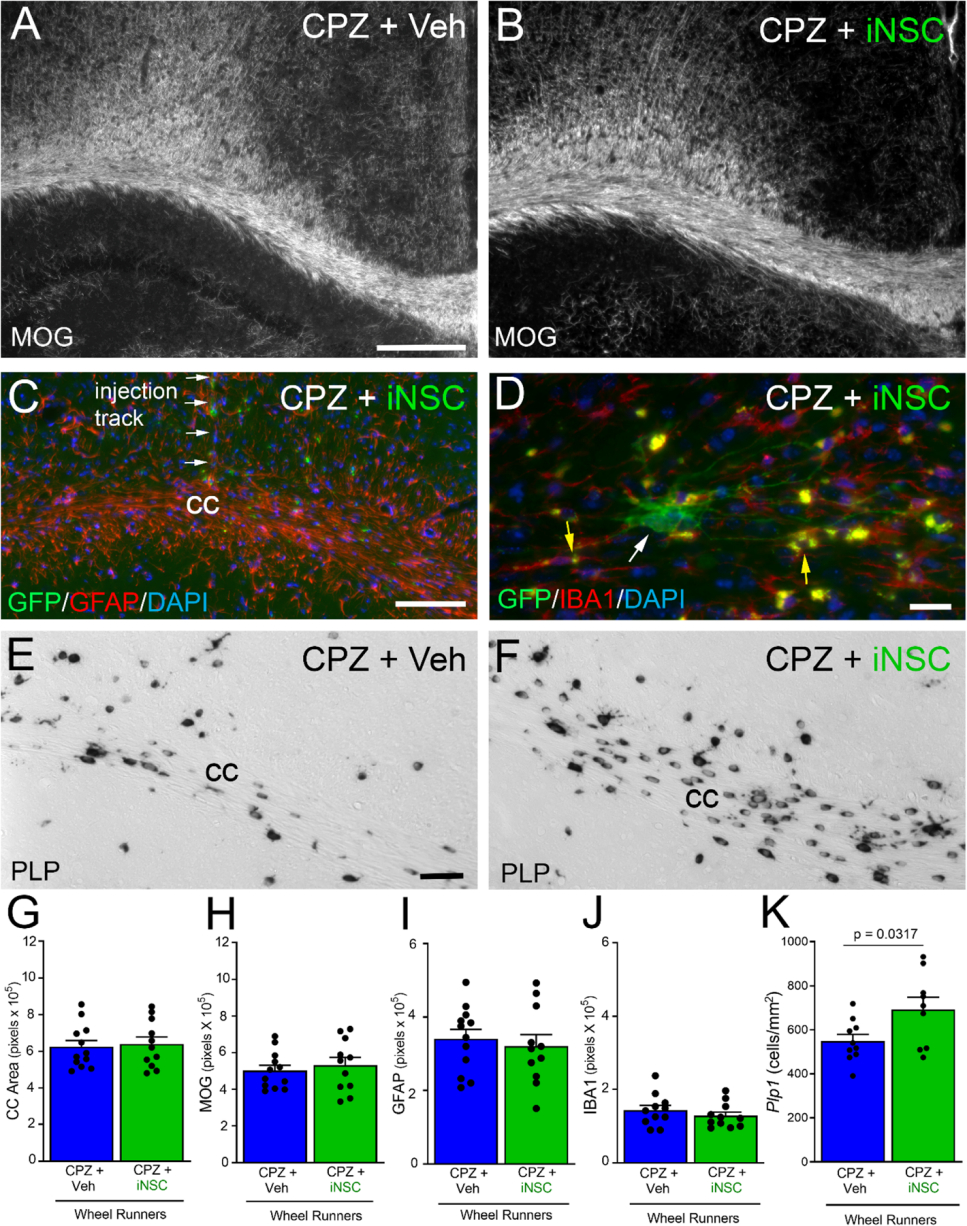
Post-behavior analysis of CC remyelination and endogenous glial cell response. **a-b:** Coronal CC sections immunolabeled for MOG to detect myelin in the wheel cohort mice after chronic CPZ with vehicle (**a**) or iNSC (**b**) injections. **c-d:** Innate immune cell response examined with immunolabeling for GFAP (**c**) or (**d**). Panel C shows astrogliosis and transplanted iNSCs (green, GFP) within the CC and adjacent areas in a section containing the region of the injection needle track. Panel D shows higher magnification of an iNSC (green, GFP; white arrow) in the CC with thin processes. IBA1 immunolabeled cells contain lipofuscin granules (yellow autofluorescence; yellow arrows). iNSCs did not immunolabel for IBA1. **e-f:** Coronal CC sections processed for in situ hybridization to detect oligodendrocytes expressing proteolipid protein (*Plp1*). *Plp1* appears upregulated after iNSCs transplantation (**f**) compared to vehicle (**e**). *Plp1* transcripts are localized in the cell body in most cells while a subset of cells show more intense labeling in the cell body and extending into processes. **g-k:** Quantification of tissue analysis in this post-behavior cohort. CC atrophy and demyelination are not attenuated by iNSCs (**g**, **h**). Transplanted iNSCs did not reduce astrogliosis (**i**) or the microglial response (**j**). In contrast, the iNSCs increased the oligodendrocyte population (**k**). Bar color reflects condition as chronic cuprizone followed by vehicle injection and wheels (blue) or iNSC transplant and wheels (green). Data are mean values ± sem. Statistical analysis used *t*-tests to compare vehicle and iNSC conditions. For immunolabeling (MOG, GFAP, IBA1), vehicle *n* = 12 and iNSC *n* = 11. For *Plp1* in situ hybridization, vehicle *n* = 10, iNSC *n* = 9. Scale bars: A, B, shown in A = 200 μm; C = 100 μm; D = 20 μm; E, F, shown in E = 50 μm

Following behavior testing, the same mice were analyzed for neuropathology and the oligodendrocyte response to iNSC transplantation (Fig. 8). Overtly, CC atrophy and myelination appeared similar in vehicle injected compared to iNSC transplant mice (Fig. 8a-b). Transplanted iNSCs were observed along the track of the injection, in the CC, cingulum, and adjacent cortex, and rarely in the underlying hippocampus (Fig. 8c). Astrogliosis persisted in the CC after iNSC transplantation and exposure to Miss-step wheels (Fig. 8c). The iNSCs did not express the microglial marker IBA1 and did not markedly alter lipofuscin granules in microglia, which were evident after chronic CPZ with and without iNSC transplantation (Fig. 8d). The oligodendrocyte populations, detected by *Plp1* expression, appeared to vary in both density and morphology (Fig. 8e-f). Note that the in situ hybridization technique for *Plp1* transcripts is not compatible with immunofluorescence detection of GFP expression to identify iNSCs in these images. Based on quantitative analyses, iNSC transplantation did not attenuate CC atrophy (Fig. 8g), demyelination (Fig. 8h), or astrogliosis (Fig. 8i), compared to vehicle injection. Microglia immunoreactivity was relatively low after the recovery period in both iNSC and vehicle mice (Fig. 8j). Importantly, iNSC transplantation significantly increased the oligodendrocyte cell density compared to vehicle injection (Fig. 8k).

Given the iNSC effect on *Plp1*, we focused on its expression during the recovery period after chronic CPZ, which appeared to vary in both density and morphology (Fig. 8e, f). *Plp1* transcripts were noted mainly in the perinuclear cytoplasm of low expressing oligodendrocytes, but also extended out into processes of oligodendrocytes expressing high levels of *Plp1* (Fig. 8f). This observation was supported by several aspects of *Plp1* expression indicating potential interest as a dynamic response during remyelination. An increase of *Plp1* expressing cells has long been associated with remyelination in MS lesions [65]. *Plp1* expression can be regulated by interaction with axons during development [66]. In neonatal rat cultures, *Plp1* transcripts are initially localized in oligodendrocyte perinuclear cytoplasm then are also present in primary processes after a delay of several days [67]. In adult mouse white matter, *Plp1* transcripts are typically localized in oligodendrocyte cell bodies [68].

**Fig. 9:**
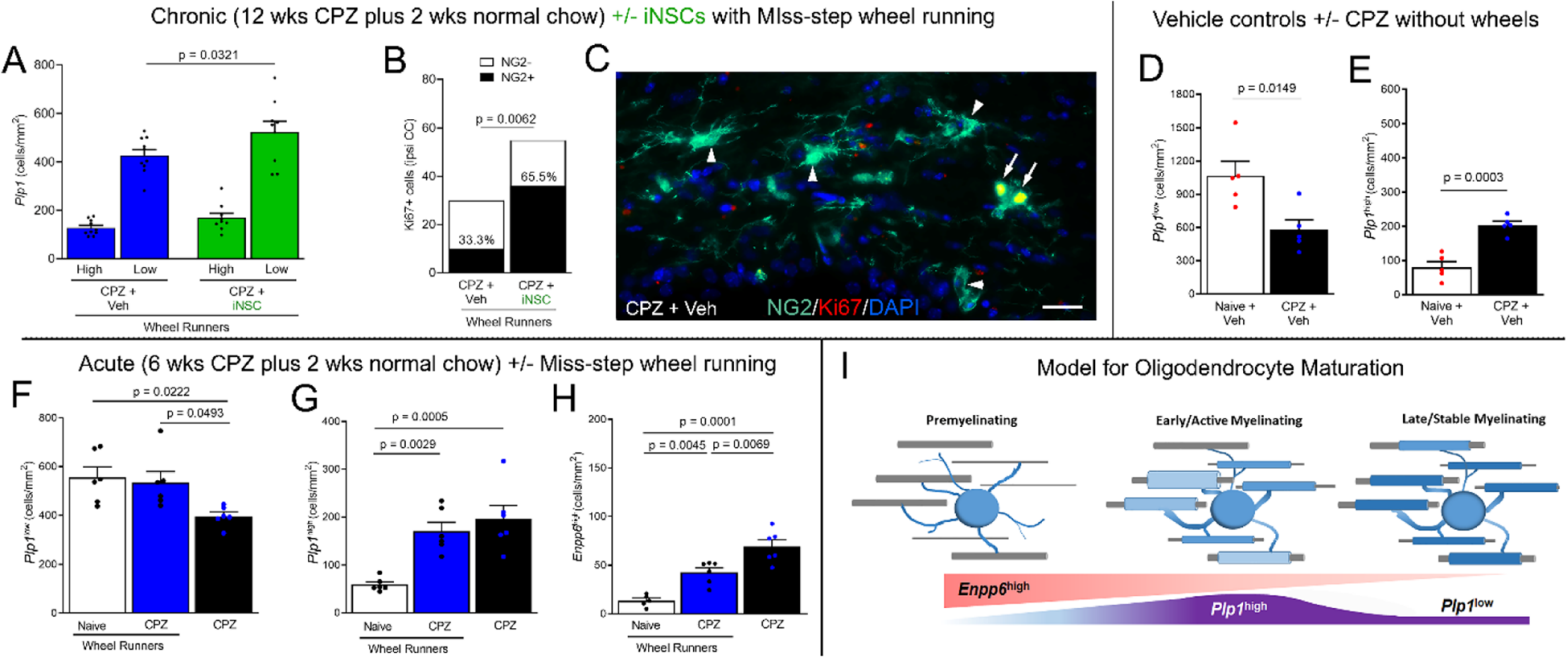
Transplantation of iNSCs alters oligodendrocyte populations during recovery. **a:** Tissues from the cohorts with Miss-step wheel access (Figs. 7 and 8) were further analyzed based on *Plp1* transcripts as either low, perinuclear signal (*Plp1*^*low*^) or high and extending into processes (*Plp1*^*high*^). iNSC transplantation increased *Plp1*^*low*^ cells during remyelination with wheel running access. **b-c:** Nuclear Ki67 identified rare proliferating cells. iNSC transplantation increased the proportion of Ki67 cells immunolabeled for the NG2, a marker of OPCs, in the corpus callosum ipsilateral (ipsi) to iNSC/vehicle injections (**b**). Example of a pair of proliferating OPCs (**c**, arrows) co-labeled for NG2 and nuclear Ki67 (overlap appears yellow) and quiescent OPCs (**c**, arrowheads). **d-e:** Analysis of additional mice without wheel access shows reduced *Plp1*^*low*^ cells (**d**) and increased *Plp1*^*high*^ cells (**e**) during remyelination after chronic CPZ. This difference indicates that *Plp1*^*low*^ and *Plp1*^*high*^ cells represent distinct lineage stages and/or responses after chronic CPZ. **f-h:** Cohorts using acute CPZ demyelination to induce extensive remyelination with and without wheel exposure. Without wheels, CPZ decreased *Plp1*^*low*^ cells (**f**) while increasing *Plp1*^*high*^ cells (**g**) as well as newly forming oligodendrocytes identified high expression of *Enpp6* (*Enpp6*^*high*^) (**h**). Miss-step wheel access during remyelination simultaneously reduced *Enpp6*^*high*^ cells and increased *Plp1*^*low*^ cells (**f**, **h**). **i:** Model integrating expression of *Plp1* and *Enpp6* transcripts during remyelination after acute and chronic CPZ with iNSC and wheel effects. For oligodendrocyte graphs, bar color reflects condition: no cuprizone (white), cuprizone without wheels (black), cuprizone with wheels (blue), and mice with iNSC (green). Data are mean values ± sem. For chronic CPZ mice from the wheel running cohort, two-way ANOVA was used with Sidak’s multiple comparisons test of CPZ vehicle (*n* = 10) versus CPZ iNSC (*n* = 9). Ki67 and NG2 data analyzed using a contingency table with Fisher’s exact test for CPZ vehicle (*n* = 3) versus CPZ iNSC (*n* = 3). Chronic CPZ mice without wheel exposure had naïve (*n* = 5) and CPZ (*n* = 5) conditions compared by *t*-tests. The acute CPZ study compared conditions of naïve (*n* = 6; *n* = 5 for *Enpp6*) and CPZ (*n* = 6) mice with wheel exposure and CPZ (*n* = 6) mice without wheels using one way ANOVA with Sidak’s multiple comparisons test

Additional analysis explored the effect of iNSC transplantation on the *Plp1* expressing oligodendrocyte populations. Oligodendrocytes were distinguished based on *Plp1* expression as *Plp1*^*low*^ with perinuclear transcript localization, or *Plp1*^*high*^ with more intense signal in the cell body and extending into processes (Figs. 8e-f and 9). Among the wheel running mice, iNSC transplantion significantly increased *Plp1*^*low*^ cells, compared to vehicle injected mice (Fig. 9a). After chronic CPZ, OPCs and Ki-67 labeled proliferating cells are relatively rare as compared to the robust proliferation and amplification of OPCs after acute CPZ [20]. However, transplantation of iNSCs increased the frequency of proliferating OPCs during remyelination with wheel access (Fig. 9b-c).

Additional studies were conducted to gain insights as to the *Plp1*^*low*^ and *Plp1*^*high*^ populations relative to demyelination and remyelination, additional mice were analyzed without wheel exposure (Fig. 9d-i). In the absence of wheel exposure, *Plp1*^*low*^ cells comprised the majority of oligodendrocytes in naïve mice and were significantly reduced after chronic CPZ (Fig. 9d). In contrast, chronic CPZ increased the *Plp1*^*high*^ population, suggesting an upregulation of *Plp1* transcription consistent with early remyelination (9E). Newly formed oligodendrocytes can be identified by high expression of *Enpp6* transcripts during learning in healthy adult mice [57]. The effect of wheel access on *Plp1* and *Enpp6* oligodendrocyte populations was examined using acute CPZ demyelination for more extensive remyelination (Fig. 9f-h). Acute CPZ reduced *Plp1*^*low*^ cells and increased both *Plp1*^*high*^ and *Enpp6*^*high*^ cells. Wheel access during remyelination simultaneously decreased *Enpp6*^*high*^ cells and increased *Plp1*^*low*^ cells. These findings can be modeled as stages of maturation during remyelination (Fig. 9i) and suggest that wheel exposure advances maturation to the *Plp1*^*low*^ phenotype.

Together, these results indicate that iNSC transplantation in combination with motor skill learning on Miss-step wheels enhanced the generation of endogenous proliferating OPCs and their differentiation into mature oligodendrocytes.

### iNSC differentiation along oligodendrocyte and astrocyte lineages exhibits interaction with host brain after transplantation into chronic lesions

**Fig. 10:**
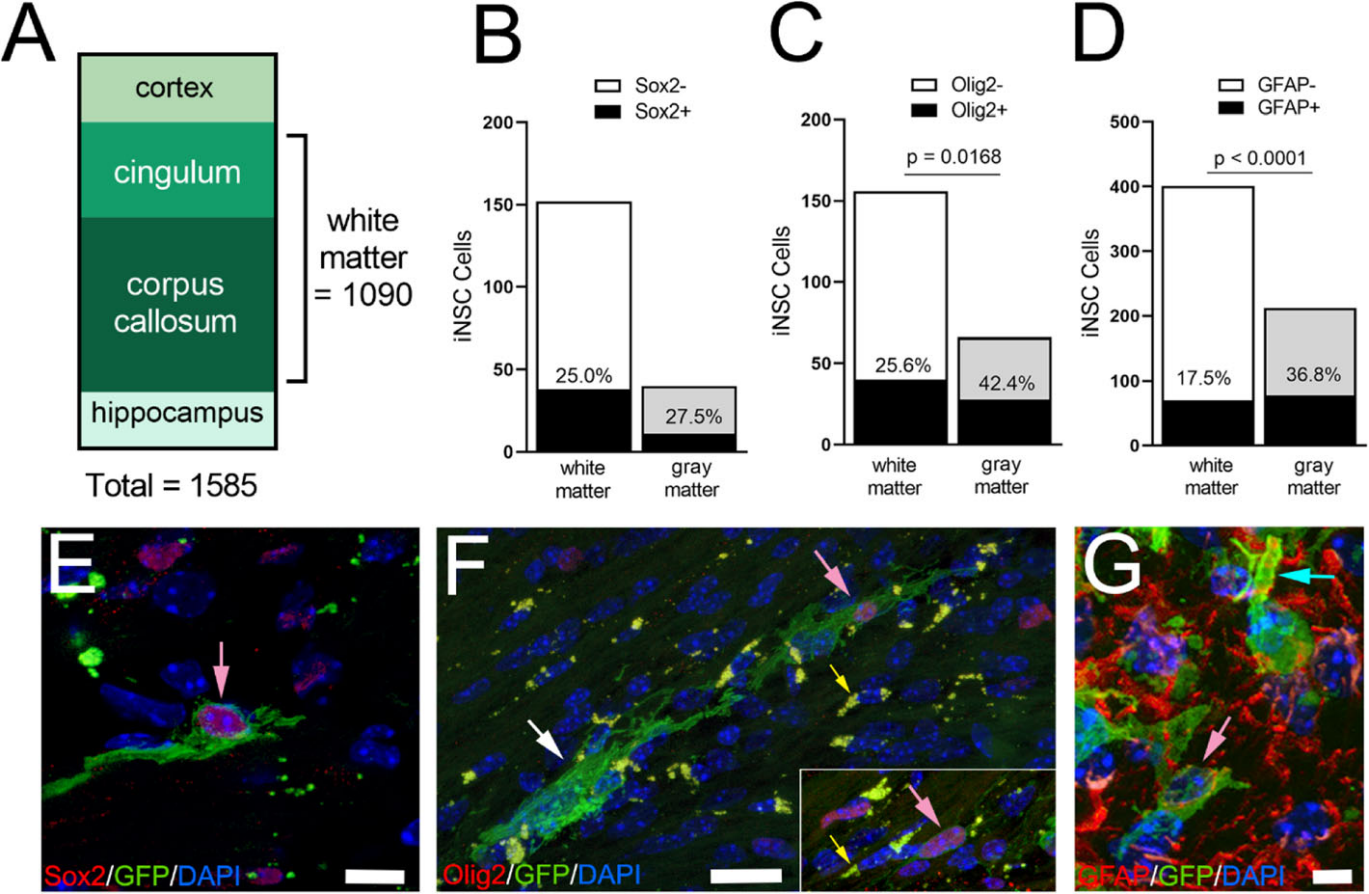
Transplanted iNSCs are localized mainly in white matter and express markers of oligodendrocyte and astrocyte differentiation. **a:** The relative distribution of iNSCs in neuroanatomical regions adjacent to the CC target site. iNSCs quantified by direct detection of GFP expression in tissue sections combined from analysis of mice in the MRI and behavior cohorts for neuropathology and iNSC cell type identification. **b-d:** Quantification of differentiation of transplanted iNSCs in white matter based on direct visualization of GFP expression and co-labeling with markers for neural stem/progenitor cells (Sox2), oligodendrocyte lineage cells (Olig2), or the astrocyte lineage (GFAP). Black fill shows counts of iNSC co-labeled for given cell marker, with upper bar section showing unlabeled iNSC counts. Percent co-labeling shown for each region. **e-g:** Examples of transplanted iNSCs expressing green fluorescent protein (GFP) along with nuclear Sox2 (**e**), nuclear Olig2 (**f**), or cytoplasmic GFAP (**g**). iNSC membranes labeled for GFP extend around cell bodies (pink arrows), along axons (white arrow), and to blood vessels (blue arrow). iNSCs were not identified as microglia in any sections analyzed with IBA1 immunolabeling. Microglia often contained autofluorescent lipofuscin granules (**f**, yellow arrows). Tissue sections from mice of both the MRI (*n* = 6) and behavior (*n* = 11) cohorts were combined for the analysis of iNSC distribution. Differentiation studies included a subset of mice (Sox2 (*n* = 6), Olig2 (*n* = 9), and GFAP (*n* = 14)) combined from MRI and behavior cohorts to generate contingency tables of total cell counts among sections analyzed for comparison using Fisher’s exact test. Scale bars E = 10 μm; F = 20 μm; G = 5 μm

iNSC location and differentiation were analyzed in mice combined from the MRI and behavior cohorts (Fig. 10). Transplanted iNSCs were located primarily in white matter of the CC or adjacent cingulum (Fig. 10a). A subpopulation of iNSCs maintained expression of Sox2 (Fig. 10b), a neural stem/progenitor cell marker [69, 70]. iNSCs differentiated along the oligodendrocyte lineage, as identified by Olig2 (Fig. 10c), and the astrocyte lineage, as identified by GFAP (Fig. 10d). In the white matter, iNSC differentiation was not significantly different between MRI and wheel cohorts for Olig2 (MRI 24.4%; wheels 24.4%; *p* = 0.8547) or for GFAP (MRI 21.2%; wheels 15.6%; *p* = 0.1673). iNSCs were also found in gray matter regions near the needle injection track (Fig. 10a-d). The tissue environment had a marked effect on the iNSC astroglial and oligodendroglial fate of transplanted iNSCs (Fig. 10c-d). iNSC differentiation into oligodendrocyte lineage cells was 31.1% O4 cells in vitro (Fig. 1d) and 25.6% Olig2 in white matter but increased significantly to 42.4% in gray matter (Fig. 10c). iNSC differentiation into astrocytes was 57.3% under in vitro conditions (Fig. 1g). Surprisingly, astrogliogenesis from transplanted iNSCs was reduced to 36.8% in gray matter and significantly decreased to 17.5% in white matter after chronic CPZ (Fig. 10d). Immunolabeling for Ki67 detected iNSC proliferation only in a neurosphere formed within the lateral ventricle under the CC in one mouse (data not shown).

The membrane-targeted GFP revealed iNSC process extension, indicating integration within the host tissue (Fig. 10e-g). Process bearing iNSCs were co-labeled for GFP along with Sox2 (Fig. 10e), Olig2 (Fig. 10f), or GFAP (Fig. 10g) cell type markers, but were not immunolabeled for IBA1 (data not shown). iNSCs were found expressing the neural stem/progenitor marker Nestin, which was not used for quantification due to immunolabeling of extensive processes of reactive astrocytes after chronic CPZ (data not shown), consistent with expression in astrocytes in reactive conditions [71]. Olig2 co-labeled iNSCs in the CC extended GFP-labeled membranes parallel to axons but did not clearly form myelin internodes (Fig. 10f), in contrast to our prior studies illustrating GFP labeling of newly formed myelin in *NG2CreERT;mTmG* mice [72]. In gray matter, GFP-labeled membranes appeared to form astroglial end feet along blood vessels (Fig. 10g).

### Axon damage after iNSC transplantation during chronic demyelination

**Fig. 11:**
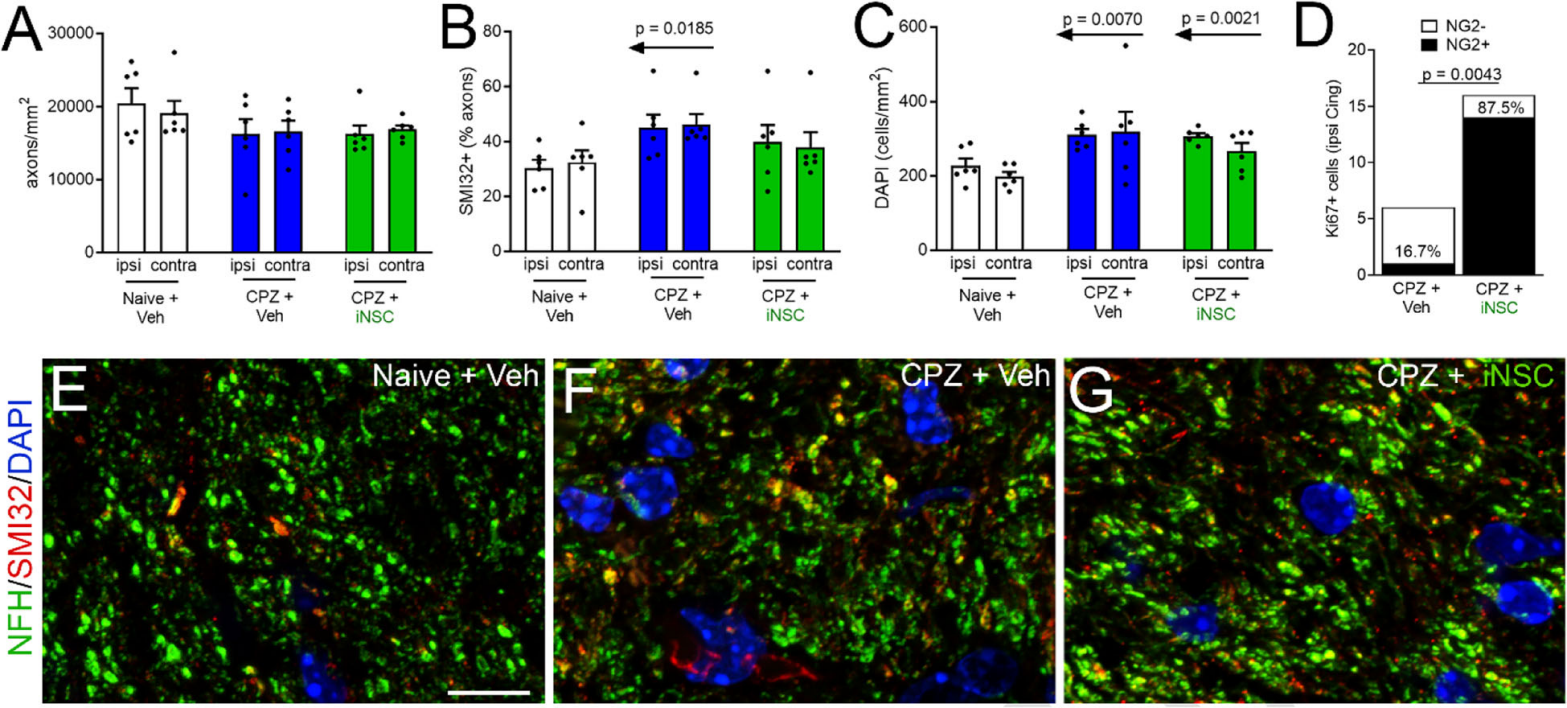
iNSC transplantation modulates axon damage and cycling OPCs after CPZ chronic demyelination in the cingulum. **a-b:** Tissues from mice with Miss-step wheel access during remyelination were further analyzed for axon damage in the cingulum to quantify the separate axon population in each hemisphere relative to vehicle and iNSC injections. Axon damage was detected by immunolabeling of total axons with neurofilament-H (**a**) and damaged axons with SMI32 for nonphosphorylated neurofilaments (**b**). Axon damage was increased significantly in chronic CPZ mice injected with vehicle while iNSC transplantation slightly improved axon health toward naïve values (**b**). **c:** Chronic CPZ increased overall cellularity in the cingulum, which was not altered by iNSC transplantation. **d:** Proliferating cells, identified by Ki67, were rare after chronic CPZ but increased with iNSC transplantation, which was largely due to an increase of NG2 immunolabeled OPCs in the ipsilateral (ipsi) cingulum. **e-g:** Representative images of each condition for neurofilament-H (pseudocolored green) labeled axons with SMI32 (red) co-labeling of damaged axons and DAPI (blue) nuclear stain. Two-way ANOVA with Sidak’s multiple comparisons test for naïve (*n* = 6), CPZ vehicle (*n* = 6), and CPZ iNSC (*n* = 6) comparison, and ipsi vs. contra comparison. Contingency table with Fisher’s exact test for comparison of Ki67 and NG2 in CPZ vehicle (*n* = 3) and CPZ iNSC (*n* = 3) mice. Data are mean values ± sem, except for contingency analysis that shows total counts for each condition. Scale bars E-G shown in E = 10 μm

Mice from wheel running studies were further analyzed to determine whether iNSC transplantation altered the extent of axon damage after chronic CPZ (Fig. 11). Axons were quantified in the cingulum which was demyelinated after chronic CPZ (Fig. 3a-b) and contained transplanted iNSCs (Fig. 10a). As compared to the CC, cingulum axons have the advantages of being distinct populations in each hemisphere that can be quantified in transverse orientation which facilitates quantification in the coronal tissue sections. Neurofilament immunostaining of the overall axon populations showed a slight reduction in the CPZ mice which was not significant (Fig. 11a). Co-immunolabeling of nonphosphorylated neurofilaments with SMI32 identifies damaged axons after chronic CPZ [41]. Naïve and CPZ mice with vehicle injections show that the injection procedure did not alter axons on the ipsilateral side (Fig. 11a-b). The proportion of damaged axons is significantly increased after CPZ in mice injected with vehicle, but not in mice with iNSC transplants (Fig. 11b). Nuclear labeling with DAPI, as an estimate of changes in cellularity, was increased after CPZ in mice receiving vehicle or iNSC transplantation (Fig. 11c). Similar to findings for the CC (Fig. 9b-c), rare proliferating cells identified by Ki67 showed a significant increase in OPCs immunolabeled for NG2 (Fig. 11d). Together, these findings indicate that exogenous iNSCs modulate multiple processes during remyelination.

## Discussion

This study evaluated the potential for transplantation of iNSCs to enhance recovery at a chronic stage of demyelination. MRI and neurological outcome measures clearly demonstrated structural and functional deficits from CPZ-mediated demyelination. MRI cohorts with iNSCs showed reduced DTI RD, a parameter often associated with aspects of remyelination after CPZ demyelination [61, 73]. Transplantation of iNSCs improved running velocities during a Miss-step wheel task, which is relevant to CC function [35].

Our study is the first to show improved performance on a neurologic outcome measure following transplantation of brain stem cells into the highly astrogliotic demyelinated lesions produced by chronic CPZ.

### Chronic CPZ model

The CPZ model is advantageous for testing cell-based therapeutics after chronic demyelination. Oligodendrocyte loss from CPZ toxicity facilitates testing cell replacement from transplanted cells. CPZ pathology is similar to MS lesions, specifically pattern III pathology with oligodendrogliopathy and low lymphocyte infiltration [18, 74–76]. CPZ produces extensive astrogliosis that persists through chronic demyelination [23]. In addition to demyelination, CPZ results in axon damage that forms varicosities from disrupted rapid axonal transport during the acute stage that progresses to neurofilament dephosporylation and reduced axon diameters after chronic CPZ [40, 41]. In patients with MS, CC atrophy correlates with functional outcomes [25, 26]. Chronic CPZ exhibits CC atrophy, without decreasing neurons in the cerebral cortex [24]. However, in the current experiments, transplantation of iNSCs improved function along with a moderate benefit on the oligodendrocyte lineage response and axon damage, while not reducing overall CC demyelination or atrophy.

### Neuroimaging

MRI distinguishes CPZ disease stages with predominant axon damage, demyelination, or remyelination [40, 77, 78]. DTI and other measures of CC integrity correlate with functional measures in MS patients [79]. Decreased DTI AD during acute CPZ (4 weeks) corresponds with axonal varicosities and increased cellularity, but is less effected with longer CPZ administration [40, 63]. DTI RD increased during CPZ demyelination, and decreased with improved remyelination in mice with genetic deletion of *Fgf2* or with estrogen receptor β ligand treatment [41, 63]. The current longitudinal studies complemented DTI with quantitative T2 and MTR. Our results agree with reports of increased DTI RD and T2 values along with decreased MTR during CPZ demyelination [40, 73]. Furthermore, mice with transplanted iNSCs had reduced DTI RD during remyelination. Although the only MRI parameter exhibiting a significant change from iNSC transplant, this difference in DTI RD has robust statistical power (96.3%) and effect size (Cohen’s *d* = 3.74). Post-imaging pathology did not detect a difference in overall CC remyelination within this MRI cohort. DTI can be influenced by cellularity or edema [64]. Therefore, iNSC effects on DTI RD may involve the reduced astrogliosis, which can contribute to local anisotropy. However, subtle changes to axon-myelin structure could also be involved yet not detected by our post-imaging analysis techniques. For example, T2 and DTI revealed abnormalities in *CST-KO* mice that have detached myelin at paranodes, without overt demyelination [80].

### Functional outcome measure

After chronic CPZ iNSC transplantation promoted recovery as detected using the Miss-step wheel running task. Miss-step wheels have a non-uniform pattern of missing rungs. Mice learn to avoid stepping faults by placing a hind paw onto a rung previously grasped by a forepaw; learning this motor skill stimulates myelination from CC oligodendrocyte lineage cells in healthy adult mice [35]. The population of newly forming oligodendrocytes, expressing *Enpp6*, indicated interactive effects of Miss-step wheels on endogenous cells during remyelination. Learning this skilled motor task may have contributed to effects on oligodendrocyte differentiation, since neuronal activity can modulate both new and existing oligodendrocytes to contribute to remyelination [81, 82]. After the learning phase, deficits in plateau running velocity correspond with myelination status [33, 35]. This Miss-step wheel running may also work as a targeted exercise enrichment, acting in conjunction with iNSC transplantation to promote recovery. For example, normal running wheels acted in parallel with clemastine, a pro-remyelinating treatment, to additively enhance remyelination after lysolecithin demyelination [15].

Together, these findings suggest an intrinsic regenerative potential for iNSC-based therapeutics and anticipate further studies to examine the interaction between targeted enrichment strategies and advanced regenerative medicines that are targeted to improve function.

### Host endogenous cell responses

The cohorts that exhibited improved function after iNSC transplantation also had increased populations of host endogenous oligodendrocytes. Transplantation of iNSCs increased the total *Plp1* expressing cells in the CC among the mice that showed improvement of Miss-step wheel running. The iNSC transplants significantly increased the *Plp1*^*low*^ population and may have transitioned from the *Plp1*^*high*^ population. The current results are the first to show that both *Plp1*^*high*^ and *Enpp6*^*high*^ cells increase during remyelination. Mice with iNSC transplantation and wheel access also had an increased proliferative response that was mainly comprised of OPCs. Thus, iNSCs may act in conjunction with Miss-step wheel running to promote differentiation of endogenous OPCs into oligodendrocytes. This interpretation is also in agreement with the slight increase of *Plp1* expressing cells after iNSC transplantation in the MRI cohort.

Chronic CPZ produced persistent CC neuroinflammation, with astrogliosis being more marked than microglial activation. Astrogliosis may mediate beneficial and detrimental effects on remyelination and axon degeneration in the progression to chronic demyelination in CPZ and in MS [23, 83, 84]. Astrogliosis was significantly reduced by iNSC transplantation in the MRI cohort. However, this GFAP reduction was not found in the wheel cohort. Therefore, interpreting effects of iNSC transplantation and/or wheel exposure may require deeper analysis of distinct astrocyte phenotypes, which have been reported to switch during early vs. late remyelination in an autoimmune model of demyelination in rats [85].

One potential explanation for these differences in *Plp1* and GFAP analyses in the MRI and wheel cohorts could be that wheel running may elicit an interaction between the iNSC transplants and the host cells or tissue environment. An alternative to consider is the potential for false positive results when the statistical power drops below the conventional 80% level [86]. Due to the death of one mouse, the power for the GFAP analysis in the MRI iNSC cohort fell to 74.4%, yet with an effect size of 1.62 (Cohen’s *d*). The increase of *Plp1* cells after iNSC transplantation in the wheel cohort was 62.3%, with an effect size of 1.09.

### Stem cell-based therapeutics

The current approach of direct conversion of mouse somatic cells, e.g. fibroblasts, into iNSCs provides stably expandable populations, and is feasible for generating human iNSCs [29–32]. Direct conversion of fibroblasts circumvents the pluripotent state, which delivers autologous NSCs more rapidly than by iPSC-type reprogramming and subsequent differentiation and is known to reduce neoplastic potential and increase genomic stability [87]. Human iNSC can be either derived by reprogramming from adult dermal fibroblasts [30] or peripheral blood monocytes [32]. Preclinical research has revealed an unexpected ability of iNSC therapies to provide neurotrophic support and inhibit detrimental host immune responses in vivo, after transplantation into the chronically inflamed CNS [88].

After transplantation into chronic CPZ lesions, iNSCs remained undifferentiated, or differentiated along oligodendrocyte or astrocyte lineages; the tissue environment significantly modulated glial differentiation (Fig. 10). iNSCs did not appear to form substantial new myelin within 2 weeks post-transplantation. Thus, iNSCs likely improved function by stimulating endogenous cell responses. As with NSCs generated by other means, iNSCs may exhibit beneficial paracrine effects on endogenous cells [89].

A recent review of cell-based therapeutic strategies for MS indicated that the preferred course of action for clinical translation may be through beneficial paracrine effects from stem cells, rather than direct cell replacement requiring injections into multiple lesion sites [88, 90]. When applied to NSC therapeutics, most preclinical studies have found cell replacement to be secondary to other “bystander” effects, in which transplanted NSCs modulate homeostasis favoring neuroprotection and immunomodulation via multiple mechanisms. NSCs in fact exert direct neuroprotective action through the secretion of neurotrophic factors; NSCs also inhibit the peripheral and perivascular activation of pro-inflammatory T cells, while increasing numbers of inflammatory T regulatory cells. In the context of progressive MS, mechanisms of action of NSCs on mononuclear phagocytes are emerging to be of pivotal importance. Among these, the reprogramming of pro-inflammatory mononuclear phagocytes through sequestration of the immunomodulatory metabolite succinate and secretion of PGE2 is common to both iNSCs and NSCs [51].

Several studies have transplanted cells into the lateral ventricle in the chronic CPZ model and reported beneficial effects without direct cell replacement. Transplanting neural precursor cells into the ventricle after chronic CPZ stimulated the endogenous OP response and improved CC remyelination [91]. Mesenchymal stem cells transplanted after chronic CPZ exhibited immunosuppressive effects on cytokines, astrocytes and microglia; this approach also activated subventricular zone NSCs and increased CC oligodendrocyte lineage cells, remyelination, and ex vivo measures of conduction velocity [92, 93]. Furthermore, NSC-Lingo-1-Fc cells, which antagonize Lingo-1, injected via tail vein during chronic experimental autoimmune encephalomyelitis effectively alleviated neurologic symptoms [94]. These findings indicate beneficial effects of stem cell transplants during chronic disease across MS models. Recently, extensive remyelination in the CPZ model was achieved with transplantation of human glial progenitor cells [95]. The human cells were transplanted during periods of OPC amplification in postnatal mice or at 4 weeks of CPZ ingestion, and so do not address transplantation into a chronic host lesion environment. Remarkably, the transplanted cells generated widespread myelin after a further 16 weeks of CPZ ingestion.

### Study limitations

Certain limitations of the current study are important to interpretation of the data. The chronic CPZ model provides a highly astrogliotic demyelinated lesion yet cannot fully replicate the pathological mechanisms of demyelination and neurodegeneration of progressive MS [76]. In addition, MS lesions are distributed within both white matter and gray matter regions [76]. Some iNSCs were localized in cerebral cortex or hippocampus, which are regions that can exhibit demyelination from CPZ [33, 96, 97], but our techniques were not designed for analysis of gray matter sites. In MS populations, sex is an important biological variable that cannot be appropriately examined in the CPZ model in C57BL/6 mice since CPZ disrupts the estrus cycle [36]. A 2-week period after transplantation was used to capture early iNSC responses in this chronic lesion environment while a longer interval could more fully examine iNSC remyelinating potential. Furthermore, comparison between mouse and human iNSC direct and/or paracrine effects will be important in future studies.

## Conclusions

These results show that iNSC transplantation can enhance recovery of function even in chronically demyelinated white matter that already exhibits significant atrophy. Our results suggest a combination of subtle beneficial effects from interactions between iNSCs and the lesion environment that promote functional recovery. As cell replacement was not required, similar effects may be achievable using small molecules and/or iNSC exosomes [89].

Altogether, despite compelling preclinical evidence of the therapeutic effects of transplantation in animal models of demyelinating diseases, further research is warranted to evaluate mechanism(s) of action from iNSC transplantation, and how to exploit these mechanisms for translation to treatments for progressive MS.
